# Patient-specific integration of RNA expression, methylation-derived replication-timing proxies, and histomorphology for pancreatic cancer subtyping

**DOI:** 10.1371/journal.pone.0358246

**Published:** 2026-09-15

**Authors:** Alejandro Leyva, M. Khalid Khan Niazi

**Affiliations:** 1 Department of Biomedical Engineering, The Ohio State University, Columbus, Ohio, United States of America; 2 Department of Pathology, The Ohio State University, Columbus, Ohio, United States of America; Huazhong Agriculture University, CHINA

## Abstract

Network biology uses gene correlations to characterize biological pathways. Linear Interpolation to Obtain Network Estimates for Single Samples (LIONESS) enables individualized gene networks, but the influence of methylation derived replication timing and tissue morphology on these networks remains unclear. We integrated replication timing proxies, RNA expression, and morphological embeddings into individualized LIONESS networks for basal like and classical pancreatic ductal adenocarcinoma using data from The Cancer Genome Atlas (TCGA). Networks and gene modules were constructed within training partitions and evaluated in held out patients using nested cross validation. The RNA expression module model achieved a mean held out area under the receiver operating characteristic curve of 0.815, comparable to the fixed Moffitt logistic regression baseline. Module selection produced reproducible RNA modules, with 18 of 29 selected genes retained in every fold, and replication timing and RNA modules, with 15 of 24 genes retained across all folds. Patient specific LIONESS edge scores did not improve subtype classification. However, replication timing and RNA integration increased global network density from 0.27 to 0.34, mean degree from 11 to 14, and transitivity from 0.64 to 0.68. Morphology and RNA integration increased density from 0.31 to 0.85, mean degree from 14 to 38, and transitivity from 0.66 to 0.92. These findings show that multimodal LIONESS networks can identify reproducible gene modules and characterize modality associated changes in biological connectivity without requiring direct mapping between separate modality specific networks.

## Introduction

Genes act in cascades regulated by intermediary messengers, such that inhibitory gene expression is inversely correlated with activating gene expression. Correlated genes within cascades can reveal mechanistic relationships among genes involved in pathways outside their immediate functional domains. Network biology provides a means to study gene coexpression by constructing networks of pairwise gene associations, with edges defined by correlation strength [1,2].

Replication timing refers to the temporal order in which genomic regions are duplicated during S phase and is closely tied to chromatin stability, accessibility, proliferation, and transcriptional potential [3,4]. Replication timing is often measured using Repli-seq, a method in which bromodeoxyuridine is incorporated into replicating DNA, enabling immunoprecipitation and quantification of newly synthesized sequences [5]. Replication timing domains are positively correlated with methylation when log-scaled, but methylation is inversely correlated with replication timing on normalized scales, indicating that highly methylated regions replicate later [6]. Because methylation correlates with replication lateness, an inverted and smoothed methylation signal can serve as a replication-timing proxy [7].

In cancer, elevated replication timing scores correlate with increased gene expression and oncogenic activity [8]. Regions harboring oncogenes such as MYC replicate earlier, reinforcing aberrant transcription in tumor cell lines [5,9]. Consequently, replication timing based on repli-seq offers a mechanistic basis for modeling differences in cascade activity and gene coexpression with respect to chromatin structure.

Pancreatic cancer includes two major transcriptomic subtypes, Basal-Like and Classical, defined by the expression of 50 genes identified by Moffitt et al. [10]. These subtypes correlate with morphological phenotypes and contain anticorrelated gene sets. Basal-Like tumors are poorly differentiated and progenitor-like, with worse prognosis and weaker response to chemotherapy, while Classical tumors exhibit epithelial features and improved treatment response [10]. Clinically, Purity Independent Subtyping of Tumors (PURIST) subtyping refines this classification using 16 of the strongest predictor genes, assessed pairwise rather than by ranked expression [11].

Gene expression has been modeled extensively in pancreatic cancer using RNA-seq, but genetic coexpression structure remains underexplored. Linear Interpolation to Obtain Network Estimates for Single Samples (LIONESS) networks enable personalized gene coexpression analysis at the patient level [12]. Although prior studies have modeled replication timing through repli-seq and gene expression jointly [3,8,13], none have done so at single-patient resolution. Moreover, no prior work has integrated morphological features extracted from whole-slide images using deep learning. By incorporating vision-transformer embeddings, genetic coexpression can be analyzed alongside physical tissue characteristics.

While RNA sequencing supports coexpression network construction, very few studies integrate replication-timing signals into multimodal networks, and none have developed individualized patient-level multimodal LIONESS networks. LIONESS supports the construction of such networks and enables mechanistic interpretation of coexpression patterns in patients [12]. Understanding these relationships allows inference of downstream gene activity within shared cascades.

Pancreatic ductal adenocarcinoma is the tenth most commonly diagnosed cancer in the United States, yet it is the third leading cause of cancer-related death. It is diagnosed late and is highly aggressive. Transcriptomic subtyping has been used to identify patients with poor prognosis, treatment resistance, and distinct cellular differentiation patterns [10,11]. Although the gene cascades underlying these subtypes are known, replication-timing contributions to subtype-specific coexpression remain unexplored. Graph-based networks allow replication-timing proxies to influence node-to-node relationships and reveal mechanistic structure in coexpression topology.

The objective of this study is to determine how methylation-based replication timing proxies and morphology influence gene coexpression structure in pancreatic cancer. We integrate replication-timing proxies, vision-transformer embeddings, and patient-level LIONESS networks to evaluate their contributions to subtype prediction and mechanistic network stability.

## Materials and methods

A total of 183 patients from TCGA PAAD were genetically subtyped using the Moffitt 50-gene classifier and mapped to methylation proxies and embeddings at a 1:1 case-to-embedding ratio [10]. All datasets are public and anonymized. For label stratification, the classical subtype was used as the positive class, while the basal subtype was used as the negative class. Methylation was preprocessed using SeSAMe and indexed using the Illumina 450K assay [6]. Raw TXT methylation files were parsed using an automatic column-detection pipeline that identified probe IDs and beta values across heterogeneous vendor formats. Beta values were converted to numeric form, clipped to the range from 0 to 1, and matched to Illumina manifest probe identifiers after normalization of probe strings. Promoter-associated probes were selected using canonical Illumina annotations (TSS200, TSS1500, 5′UTR, and First Exon), and probes mapping to multiple genes were expanded so that each gene received its corresponding probe values. No samples were excluded from any modality. Modalities were matched by linking each sample file ID to its unique patient case ID; therefore, each patient had one whole-slide image (WSI), one methylation array, and one RNA-sequencing array. For each sample, promoter beta values were averaged across all probes assigned to each gene to produce a reproducible promoter-level methylation estimate [6]. Gene-wise z-scores were computed across samples to normalize differences in probe density and distribution. Methylation states were categorized as hypomethylated, baseline, or hypermethylated using fixed z-score thresholds. Both long (sample × gene) and wide (gene × sample) methylation matrices were generated for downstream network analysis. This procedure ensured consistent promoter-level aggregation and reproducible methylation-derived replication-timing proxies across the cohort.

Methylation was converted into a replication timing proxy by inversion, z-scoring, and smoothing, following the established correlation between methylation and replication-timing domains [6,7].


$$\begin{array}{c} RT_{w}=-\text{zscore}\left(M_{w}^{\text{smooth}}\right) \end{array}$$ (1)

Because the beta matrices had already been preprocessed, smoothing was then applied across domains to normalize methylation.


$$\begin{array}{c} M_{w}^{\text{smooth}}=\text{Smooth}\left(\left\{{\tilde{M}}_{w^{\prime }}:w^{\prime }\in \text{chr}(w)\right\}\right) \end{array}$$ (2)

$$\begin{array}{c} {\tilde{M}}_{w}=\text{median}\left\{M_{i}:x_{i}\in w\right\} \end{array}$$ (3)

After smoothing, the methylation domains were z-scored such that regions with higher beta values had lower replication-timing proxy values, and vice versa. The methylation-based replication timing proxy for each domain was calculated as a length-weighted average of the inverted beta values:


$$\begin{array}{c} RT_{D}=\frac{\sum_{w\subset D}\text{𝓁}(w)RT_{w}}{\sum_{w\subset D}\text{𝓁}(w)} \end{array}$$ (4)

where ℓ(w) is the length of genomic window w. For nonuniform bins, the window length was calculated using the following equation:


$$\begin{array}{c} \text{𝓁}(w)=x_{\text{end}}(w)-x_{\text{start}}(w) \end{array}$$ (5)

For uniform bins:


$$\begin{array}{c} \text{𝓁}(w)=L=\text{2}\text{0}\text{0},\text{0}\text{0}\text{0}\text{ bp} \end{array}$$ (6)

The sample-level average methylation-based replication timing proxy and the length-weighted fraction of late-replicating domains were calculated as follows:


$$\begin{array}{c} \overset{―}{RT}=\frac{\sum_{w}\text{𝓁}(w)RT_{w}}{\sum_{w}\text{𝓁}(w)},\;\;{\text{frac}}_{\text{late}}=\frac{\sum_{w}\text{𝓁}(w)1\left\{RT_{w}>\text{0}\right\}}{\sum_{w}\text{𝓁}(w)} \end{array}$$ (7)

After the replication-timing domains were divided into 200-kb regions, networks were constructed from gene correlations to create a global correlation network across all patients. Gene correlations within replication-timing domains and replication lateness influenced edge strength and connectivity. The network-construction parameters are shown in Table 1. The minimum absolute Spearman correlation required between any two expressed genes was 0.5; a lower threshold caused the network to become oversaturated with edges. After all edges were calculated, the 5,000 largest edge weights were retained, and only the top 500 weighted edges were plotted. A minimum of five samples with non-missing values was required for each gene to be included in network parameterization. A random seed was used to ensure reproducible results.

**Table 1:** Key parameters used for individualized replication–timing (RT) and RNA network generation.

Parameter	Value
Absolute minimum correlation	0.5
Global top–K edges	5000
Plot top–K edges	500
Minimum non–missing samples	5
Random seed	1337
Replication–timing/ RNA mix	1.0, 0.5, 0.5
Number of RT shuffles	100
Grid search range	0.5:1.5:0.25, 0:1:0.25, 0:1:0.25
Partial correlation used	1
Bootstrap iterations	50
Bootstrap fraction	0.8
Top–K for stability	10000
Replication–timing threshold	0.0

The RNA-mix values are the scaling coefficients a, b, and γ used for the subtyping task. A total of 100 shuffles were performed by permuting the labels to create null-control graphs, ensuring that the observed variation was not caused by dataset artifacts. For each sample, a, b, and γ were varied to visualize changes in network behavior and saturation during the subtyping task. Only one partial-correlation matrix of the replication timing proxy and RNA was generated. Fifty iterations were used to estimate edge stability, with 80% of the networks sampled in each bootstrap iteration. The top 10,000 edges were used to compute Jaccard stability across networks. The replication-timing threshold was set to zero to visualize changes in network behavior after the methylation-based replication timing proxy was introduced.

Gene associations were established using the Spearman correlation between each pair of expressed genes. The optional partial-correlation network was created using the inverse of the gene-expression covariance matrix, denoted Ω. This matrix was used to calculate the partial correlation between two expressed genes, denoted ρ.

### Gene associations


$$\begin{array}{c} r_{ij}=\text{corr}\left(X_{i},X_{j}\right),\;\;\rho_{ij}=-\frac{\Omega_{ij}}{\sqrt{\Omega_{ii}\Omega_{jj}}},\;\Omega =\Sigma^{-\text{1}} \end{array}$$ (8)

Replication-timing correlations were derived from the average methylation proxied replication timing of each domain. Here, i and j denote genes, while u and v represent all genes within the domains containing i and j.

### Replication-timing similarity


$$\begin{array}{c} s_{ij}^{\text{RT}}=\text{1}-\frac{\left|RT_{D}(i)-RT_{D}(j)\right|}{{\text{max}}_{u,v}\left|RT_{D}(u)-RT_{D}(v)\right|} \end{array}$$ (9)

Edge weights between gene pairs were influenced by the similarity of their RT domains, their gene-expression correlation, and the z-score-normalized gene-pair correlation. This produced an RT-influenced RNA graph that integrated replicative domains with correlations between genes in the corresponding replication domains.

### Integrated edge weights


$$\begin{array}{c} w_{ij}^{\text{RNA}}=r_{ij}\text{or}\rho_{ij},\;\;{\tilde{s}}_{ij}^{\text{RT}}=\text{2}s_{ij}^{\text{RT}}-\text{1} \end{array}$$ (10)

For each gene pair, the integrated RT-RNA network used the Spearman correlation of expression and the Spearman correlation of replication-timing profiles. Replication-timing values were rank-transformed before correlation.


$$\begin{array}{c} w_{ij}^{\text{INT}}=a\;w_{ij}^{\text{RNA}}+b\;{\tilde{s}}_{ij}^{\text{RT}}+\gamma \;z\left(w_{ij}^{\text{RNA}}\right) \end{array}$$ (11)

with mixing coefficients a,b,γ selected from the grid ranges reported in the Methods.

Edge weights were thresholded using τ, with the absolute-correlation cutoff reported in the methods table (r = 0.5). The top-K edge weights specified in the parameter table were then used to construct the global network. Patient-specific networks were estimated with LIONESS using the RT-RNA integrated-similarity function; RNA-only and RT-only networks were analyzed only at the global level.

### Edge selection


$$\begin{array}{c} W^{⋆}=\left\{(i,j):\left|w_{ij}^{\text{INT}}\right|\geq \tau \right\},\;\;W^{\text{top}K}=\text{TopK}\left({\left\{\left|w_{ij}^{\text{INT}}\right|\right\}}_{(i,j)\in W^{⋆}},K\right) \end{array}$$ (12)

LIONESS networks were constructed to generate personalized networks for each patient. Patient-specific edge weights were computed from the difference between each edge in the global network and the corresponding edge calculated after excluding that patient.

### Individualized networks (LIONESS)


$$\begin{array}{c} e_{ij}^{\left(\text{all}\right)}=\text{edge from all }N\text{ samples},\;\;e_{ij}^{\left(-q\right)}=\text{edge from all samples except }q \end{array}$$ (13)

$$\begin{array}{c} e_{ij}^{\left(q\right)}=N\;e_{ij}^{\left(\text{all}\right)}-(N-\text{1})e_{ij}^{\left(-q\right)} \end{array}$$ (14)

Module scores quantified the mean personalized edge weight within each gene cluster, using the patient-specific weights e(q) for edges belonging to module Eₖ.

### Module scores


$$\begin{array}{c} m_{k}^{\left(q\right)}=\frac{\text{1}}{\left|E_{k}\right|}\sum_{(i,j)\in E_{k}}e_{ij}^{\left(q\right)} \end{array}$$ (15)

A sigmoid function was applied to a linear projection of the module scores and learned coefficients βₖ to predict the basal or classical subtype for each patient. This allowed the model to identify the gene clusters most strongly influenced by the replication timing proxy. A sample was classified as basal when its predicted basal-subtype probability exceeded 50%.

### Subtype prediction


$$\begin{array}{c} {\hat{p}}_{\text{basal}}^{\left(q\right)}=\sigma \left(\beta_{\text{0}}+\sum_{k}\beta_{k}m_{k}^{\left(q\right)}\right),\;\;{\hat{y}}^{\left(q\right)}=I\left[{\hat{p}}_{\text{basal}}^{\left(q\right)}\geq \text{0}.\text{5}\right] \end{array}$$ (16)

where $\sigma (z)=\frac{\text{1}}{\text{1}+e^{-z}}$. Any linear or margin-based classifier applied to $\left\{m_{k}^{\left(q\right)}\right\}$ is equivalent within this framework.

For a real-data metric, the Area Under the Receiving Operating Curve (AUC), connectivity, or correlation—Z-score significance was calculated relative to the shuffled modules. Here, μ is the mean and σ is the standard deviation of the shuffled-module metrics. The empirical p-value was calculated as the proportion of shuffled metrics greater than or equal to the real value, with a plus-one correction. Jaccard stability was computed across bootstrap edge sets A and B, and the expected value was taken across distinct bootstrap pairs [14–16].

### Null comparison and stability


$$\begin{array}{c} Z_{\text{metric}}=\frac{x_{\text{real}}-\mu_{\text{shuffle}}}{\sigma_{\text{shuffle}}},\;\;p_{\text{emp}}=\frac{\text{1}+\#\left\{x_{\text{shuffle}}^{\left(s\right)}\geq x_{\text{real}}\right\}}{\text{1}+S} \end{array}$$ (17)

$$\begin{array}{c} \text{Jaccard}(A,B)=\frac{\left|A\cap B\right|}{\left|A\cup B\right|},\;\;\text{Stability}=E_{b_{\text{1}}\text{≠}b_{\text{2}}}\left[\text{Jaccard}\left(W_{b_{\text{1}}}^{\text{top}K},W_{b_{\text{2}}}^{\text{top}K}\right)\right] \end{array}$$ (18)

Using the same network-construction framework, morphology–RNA networks were generated from 1,536-dimensional UNIv2 patch embeddings extracted through Trident at 20 × magnification. Tissue segmentation was performed using HEST to isolate tissue regions and remove artifacts. Each 256 × 256 patch was processed using 3 × 3 convolutions and then passed through a pretrained UNIv2 encoder to produce the patch embeddings. Patch embeddings from each slide were averaged to obtain a patient-level morphological feature vector. Each patient had a slide-level representation of their patches that was matched to the other modalities using the patient case ID. All cases were surgical resections from primary tumors. These features were standardized and incorporated into the module-scoring stage of the network pipeline, where morphology was used to weight module activation and influence the gene clusters selected for subtype prediction. Morphology was not used to create separate morphological nodes; rather, the morphological representation modulated the scoring of RNA-derived modules within each patient’s LIONESS network. All embeddings were extracted using the same preprocessing parameters to ensure reproducibility. Partial correlation was not used to construct the networks, but one network was included as a control experiment. Diagram 1 shows the overview of the network dynamics process and preprocessing methods.

Diagram 1: An overview of the study, whereby RNAseq, methylation, and whole slide histology are used and preprocessed to produce expression graphs that are influenced by each modality.

The model is trained using stratified five-fold cross validation 80/20 train-test. The cross validation is trained such that the feature/module selection steps were repeated independently within each training partition, and tested on an outer partition of the training set. Once the gene module is established and fitted onto a regression, the gene module is tested on an outer-test fold outside of the training fold. Once the gene module is finished being constructed within each outer training-partition, each fitted score regression is parameterized within the four training folds are split into three inner cross validation folds, whereby the gene modules are chosen using top k edges using the process, and the module scores are computed and used to validate the gene module in the third outer fold within the four train folds. The outer test fold was never used for module construction or tuning. The gene modules are evaluated through performance by AUC, whereby AUC was calculated from the continuous module scores with classical patients defined as the positive class. Values below 0.5 indicate that higher module scores were associated with the basal-like subtype. For the nested crossfold-validations, once the gene module is validated within the three folds, then the module scores are applied to fit a regression, which is then applied to the outer test fold. This regression is used to generate the threshold for subtyping. In total, there are 15 different gene modules being sampled per run. The null gene modules and graphs are used to test the parametrizations of the graphs and to see whether the correlational edges impacted performance.

Unless otherwise stated, all AUCs were calculated with classical as the positive class and basal-like as the negative class. An AUC below 0.5 therefore indicates that the continuous score is oriented in the basal-like direction, rather than an effect of class imbalance. Where basal-positive AUC is reported, it was calculated as 1−AUC from the same scores and is provided only to show score direction.

## Results

Preprocessing, network construction, and statistical validation were performed on the Ohio State Supercomputer using NVIDIA A100 Graphics Processing Units (GPUs). The scripts required 30 minutes to assemble the networks and perform subtyping.

The number of Early-Early (EE), Early-Late (EL), and Late-Late (LL) replication-timing gene pairs was measured for each variable. Jaccard stability remained high despite the small gene set and large number of bootstrap iterations. Density and transitivity plots showed that the shuffled results clustered together, demonstrating that the variance was reproducible and was not caused by dataset artifacts. All network conditions were constructed using the complete matched Cancer Genome Atlas – Pancreatic Adenocarcinoma (TCGA-PAAD) study cohort and were not stratified by subtype. RNA denotes RNA coexpression networks; RT denotes methylation-derived replication-timing-proxy networks; RT–RNA denotes integrated replication-timing-proxy and RNA networks; RNA-PCORR and RT–RNA-PCORR denote their partial-correlation variants; MORPH–RNA denotes morphology-influenced RNA networks; GRID denotes grid-search parameterizations; and SHUFFLE denotes label-permuted null-control networks.

**Fig 1:**
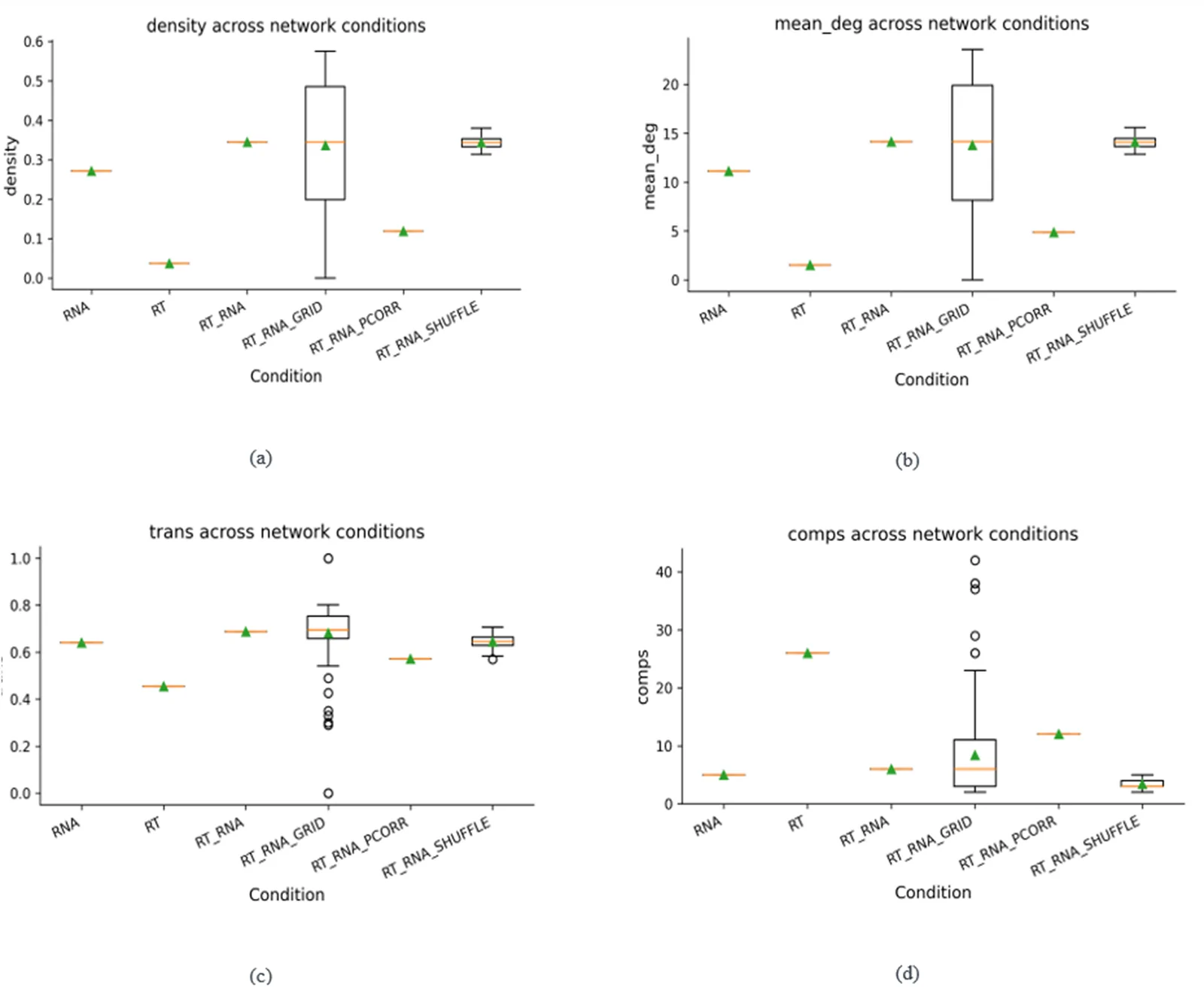
Global network topology across conditions. Boxplots show (a) network density, (b) mean node degree, (c) transitivity, and (d) the number of connected components for the RT, RT_RNA, RT_RNA_PCORR, grid search, and shuffled control networks.

Fig 1 shows the global network characteristics across conditions.

**Fig 2:**
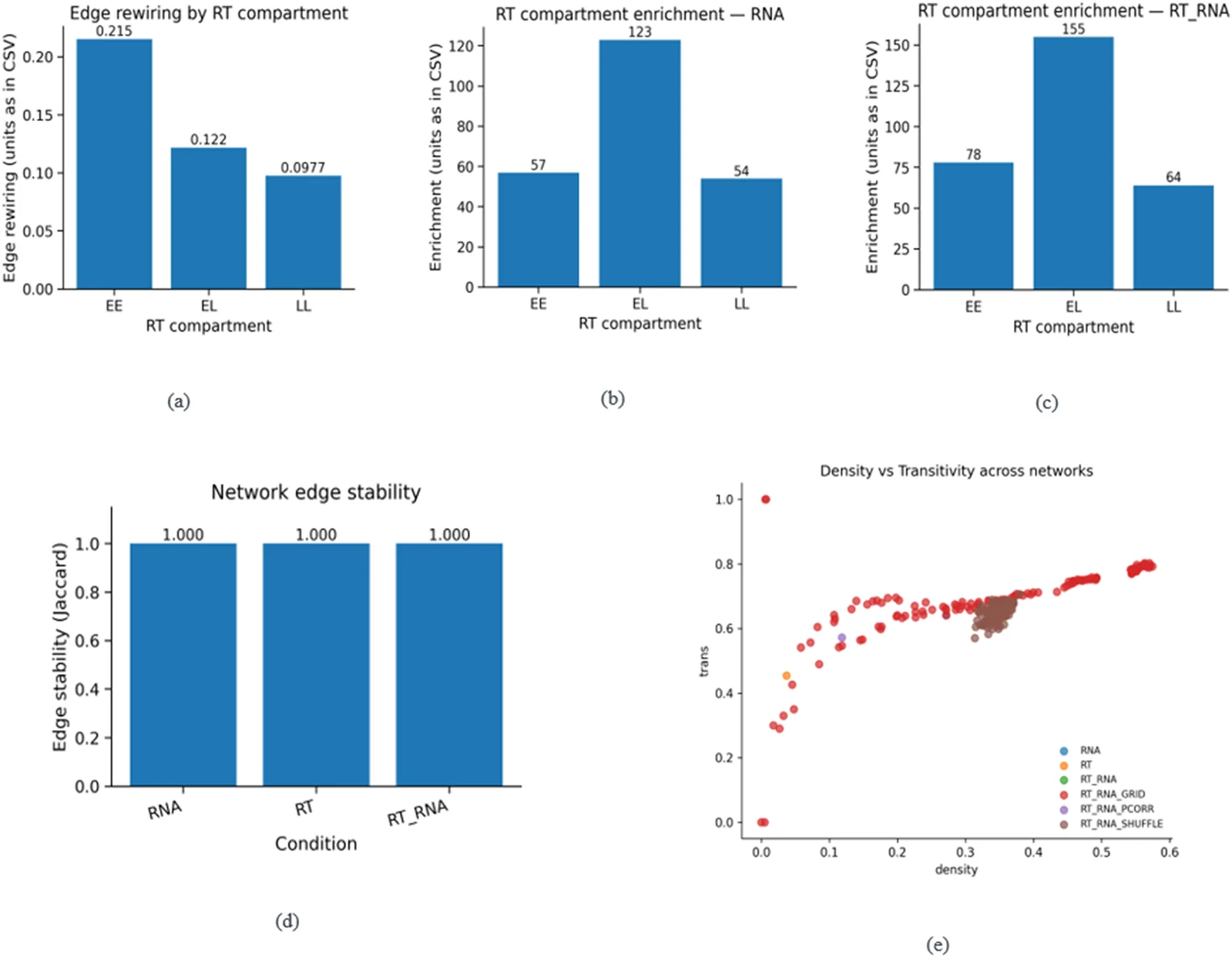
Replication-timing context and stability. (a) Edge rewiring across early and late replication timing compartments. Replication timing compartment enrichment is shown for (b) the RNA condition and (c) the RT_RNA condition. (d) Edge stability across network conditions. (e) Relationship between network density and transitivity.

The results in Tables 2 and 3 demonstrate that the individualized networks provided insufficient subtype discrimination with the 50-gene system, with signals barely above noise. RT domains could not be modeled as individual nodes because each domain encompassed multiple genes. Fig 2 shows the RT network characteristics with bulk RNA.

**Table 2:** Subtype-classification performance (AUC) across conditions for basal-like and classical pancreatic ductal adenocarcinoma using a single global graph across patients using degree based module scores from Moffitt genes. The absolute correlation threshold was set to 0.2 to measure performance with the maximum number of valid degrees. In turn, retaining a larger number of lower-weight edges altered the degree-weighted scores among patients, whereas limiting the number of retained edges could reduce score variance across graph conditions because the graphs differed in their edge-weight distributions and edge counts. For the global-graph analysis, lowering the threshold allowed us to evaluate how heterogeneous connectivity and increased variation in degree-weighted patient scores affected model performance. All other experiments set the edge correlation threshold to 0.5 to isolate strongly correlated subsets of genes.

condition	Subtype AUC
RNA	0.629
RT	0.619
RT_RNA	0.636

AUC values summarize discrimination between basal-like and classical subtypes using the specified network inputs as an exploratory study. The reported columns are preserved exactly from the source data.

**Table 3:** Global module-level AUCs derived from mean-based replication-timing–proxy features alone as a module level ablation study.

module	No. genes	AUC
module_0	6	0.537037
module_2	4	0.474074
module_3	3	0.470370
module_1	5	0.387963

These exploratory results isolate the contribution of replication-timing proxies to subtype prediction independently of RNA as an exploratory result.

**Fig 3:**
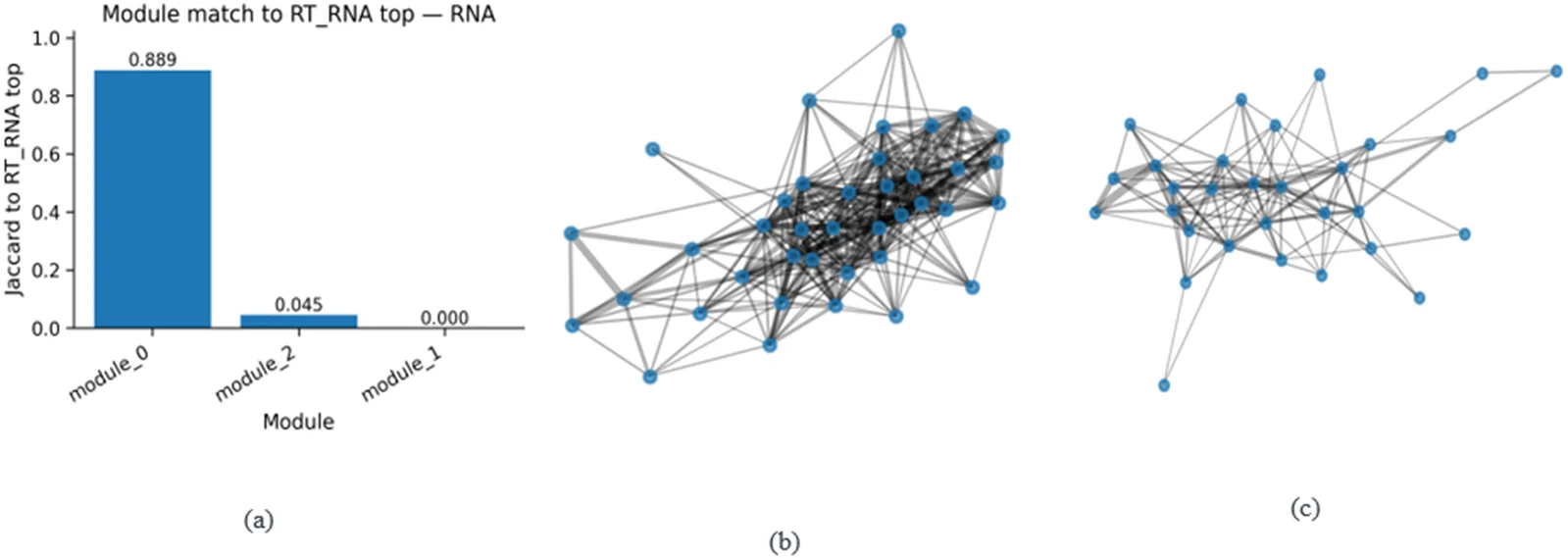
(a) Module-level Jaccard overlap with the top RT-RNA modules. Bars show the similarity of each module, highlighting modules stabilized or strengthened when replication-timing proxies were integrated. (b) Global and (c) patient-specific networks for the RT/RNA conditions are also shown.

The module-level predictions demonstrate that the RNA-only and integrated RT-RNA modules achieved similar performance using the same 17 genes shown in Tables 4 and 5 within a single gene-cluster module. The 17 genes are listed in Table 6. The peak AUC of 0.8 across the module-level conditions, together with the increased edge correlations, indicates that adding RT increased network robustness while maintaining the same performance. The methylation-based replication timing proxy provides a correlative view of gene coexpression mechanisms but did not improve output accuracy, which depended on the strength of the gene clusters. Fig 3 shows the network visualizations for the RT-RNA networks.

**Table 4:** Module-level AUCs derived from mean based RNA-only global networks. These results show which modules are most informative for subtype prediction as an ablation study.

module	No. genes	AUC
module_0	17	0.805688
module_2	6	0.595767
module_1	15	0.380820

This table highlights the RNA-only network modules that contributed to classification and should be interpreted alongside the RT-only and RT + RNA module tables to compare signal localization as an exploratory result.

**Table 5:** Module-level AUCs from joint replication-timing proxy and RNA networks using LIONESS mean specific edge weights, representing the integrated model. Columns are preserved exactly as provided.

module	No. genes	AUC
module_1	17	0.789418
module_0	20	0.426852

These values can be compared with the RNA-only and RT-only results to identify modules in which integration improved stability or performance.

**Table 6:** Mapped genes for module 1 with HUGO Gene Naming Committee (HGNC) symbols and descriptions.

Gene	Ensembl ID	Description
LAMC2	ENSG00000058085	laminin subunit gamma 2
COL17A1	ENSG00000065618	collagen type XVII alpha 1 chain
DHRS9	ENSG00000073737	dehydrogenase/reductase 9
SLC2A1	ENSG00000117394	solute carrier family 2 member 1
TNS4	ENSG00000131746	tensin 4
SCEL	ENSG00000136155	sciellin
TMPRSS4	ENSG00000137648	transmembrane serine protease 4
MAL2	ENSG00000147676	mal, T cell differentiation protein 2
SPRR3	ENSG00000163209	small proline rich protein 3
SPINT2	ENSG00000167642	serine peptidase inhibitor, Kunitz type 2
KRT19	ENSG00000171345	keratin 19
CST6	ENSG00000175315	cystatin E/M
LEMD1	ENSG00000186007	LEM domain containing 1
S100A14	ENSG00000189334	S100 calcium binding protein A14
S100A2	ENSG00000196754	S100 calcium binding protein A2
LAMB3	ENSG00000196878	laminin subunit beta 3
KRT6A	ENSG00000205420	keratin 6A

**Fig 4:**
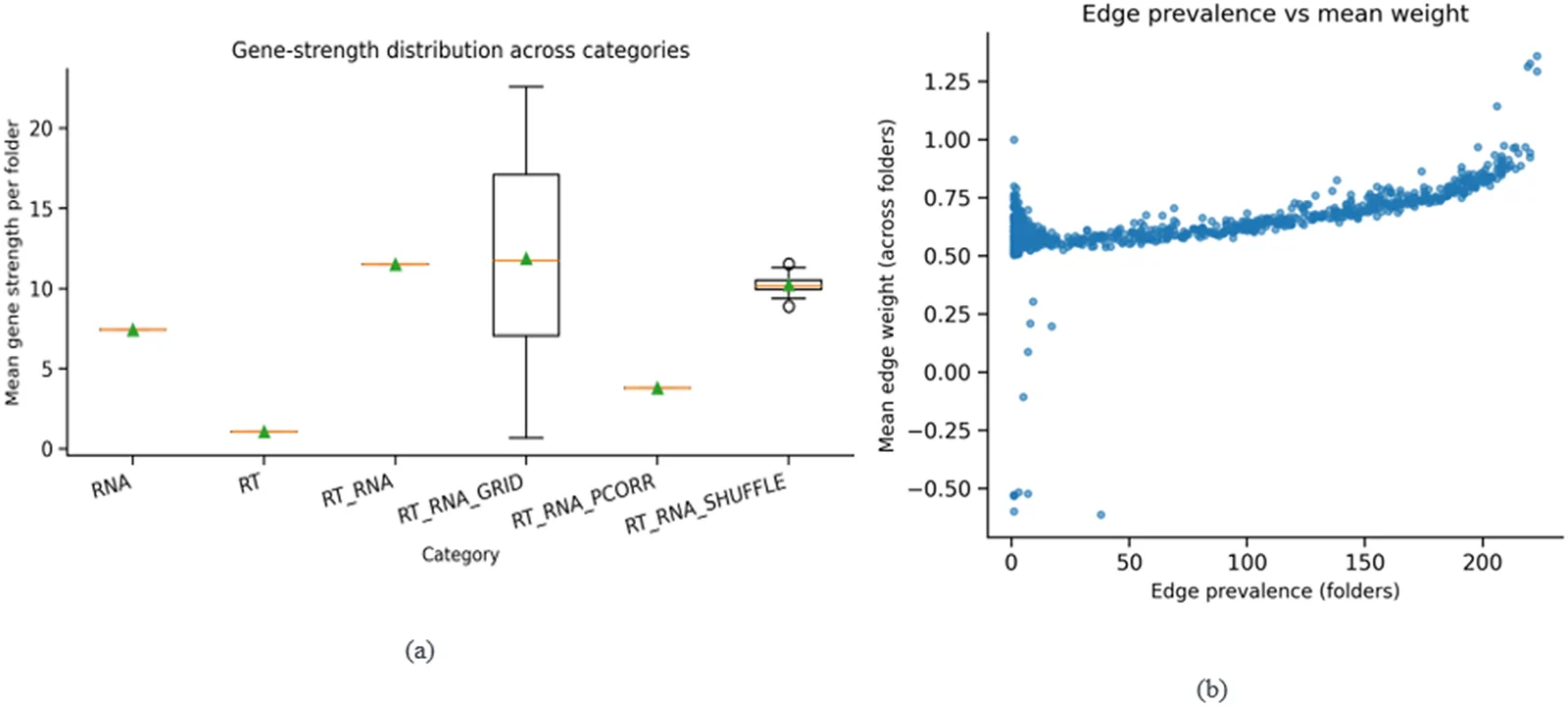
(a) Gene strength by condition; (b) Edge prevalence versus mean edge weight for each condition.

Fig 4 shows gene-correlation strength across the network conditions, including grid conditions with altered parameters.

**Fig 5:**
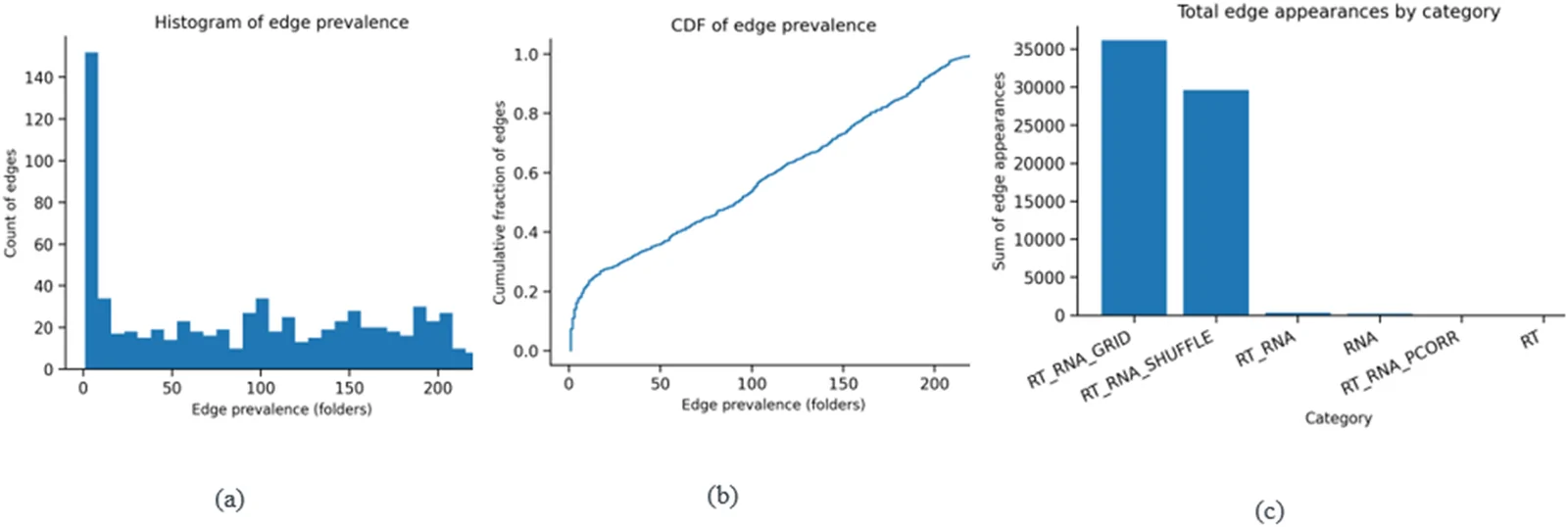
Distribution and frequency of edge weights across network construction conditions. (a) Histogram of edge prevalence showing how often individual edges appear across RNA, RT, grid-search, and shuffle network variants. (b) Cumulative distribution function of edge weights illustrating the overall edge-weight distribution after thresholding and top-K selection. (c) Total number of edge appearances aggregated by network category, highlighting the larger edge counts produced by grid-search and shuffle conditions compared with the constrained global RNA and RT-RNA networks.

The RT and RNA enrichment results showed a natural overlap of gene sets across the RT and RNA conditions. Specifically, the gene sets overlapped by 88% across conditions. One shuffled global network is shown for the RT and RNA conditions alongside a LIONESS network. These networks displayed consistent edges without anomalous or obscure connections. Fig 5 shows the distribution and occurrence of edges within each condition after correlation filtering.

Across patient-specific networks, edges with higher weights were more prevalent, supporting the use of edge weights to evaluate gene-correlation strength across clusters. Across conditions, the RNA grid search showed the greatest variability, while the shuffled control networks showed little shift and stable overlap. Table 6 shows that 2 genes in module 0 of the RT and RNA modules overlap with the PURIST gene set, 9 of said genes overlap with the basal-like genes, which has a reported subtyping AUC of 99%. Other genes including GATA6 and REG4 are markers of classical lineage. This overlap is partly the result of random assortment, but also due to the optimization of the gene modules in the fold validation, resulting from rederivation from moffitt and other genes. In this case, subtyping accuracy depended on gene-cluster strength rather than global network strength. Fig 5 shows a large number of low-weight edges that were filtered from the networks. As expected, the RNA and RT grid-search and shuffled networks contained more edges than the constrained global RNA + RT networks. Partial correlation was not used, and RT alone could not generate edge weights because it contained no gene-specific weights.

**Fig 6:**
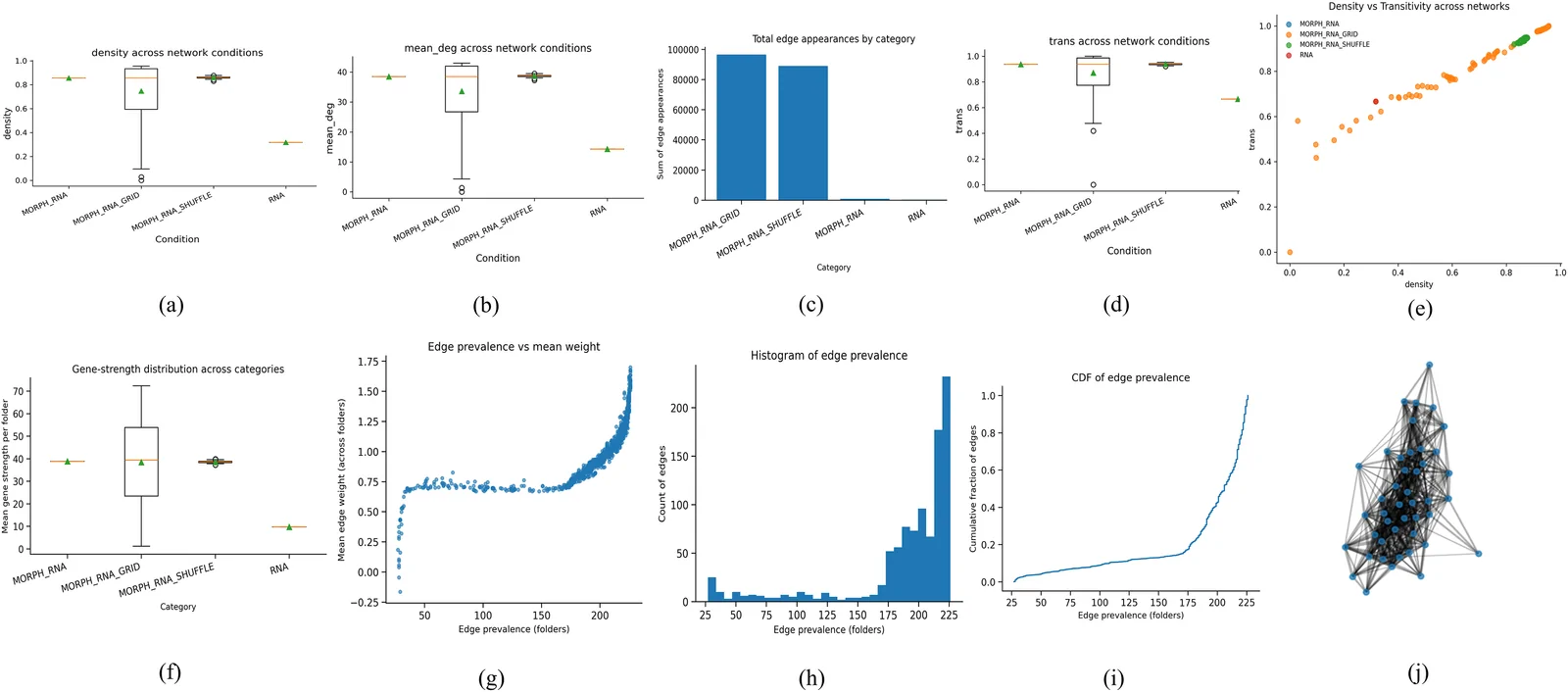
(a) Network density across the MORPH_RNA, MORPH_RNA_GRID, MORPH_RNA_SHUFFLE, and RNA conditions. (b) Mean node degree across network conditions. (c) Total number of edge appearances aggregated within each condition. (d) Network transitivity across conditions. (e) Relationship between netfwork density and transitivity for individual network configurations. (f) Distribution of mean gene strength across conditions. (g) Relationship between edge prevalence, defined as the number of network configurations containing an edge, and the mean weight of that edge across configurations. (h) Distribution of edge prevalence. (i) Cumulative distribution of edge prevalence. (j) Global morphology influenced RNA network, with nodes representing genes and edges representing weighted gene associations. Boxplots show the median as an orange line, the mean as a green triangle, and outlying values as open circles. All conditions were constructed using the complete matched TCGA PAAD study cohort and were not stratified by subtype. MORPH_RNA denotes the morphology influenced RNA network, MORPH_RNA_GRID denotes grid search parameterizations, MORPH_RNA_SHUFFLE denotes shuffled control networks, and RNA denotes the RNA coexpression network. The panels present descriptive network statistics, and no inferential hypothesis test was applied.

Morphology-RNA networks constructed using UNIv2 embeddings influenced module scores and edge weights, producing a skewed distribution of edge variance. Shuffled controls indicated that this variance was not attributable to dataset artifacts (Fig 6). Because only one global morphology-RNA graph and one global RNA graph were constructed, their total edge appearances were lower than those aggregated across the grid search and shuffled conditions. Mean degree varied substantially across the grid search, whereas the RNA networks remained stable. Edge prevalence was relatively stable as a function of mean weight, although its distribution was left skewed. The global morphology-RNA network is shown in Fig 6j. Overall, morphology based networks derived from vision transformer embeddings were stably associated with gene coexpression within the Moffitt gene cohort [1,2,10,12].

Tables 7–9 show that the morphological LIONESS networks exhibited a dipole-like shift, whereby morphological embeddings preferentially emphasized one subtype and selected genes corresponding to modules related to that subtype. Module 0 showed a strong basal-subtype preference, with its AUC reduced by ambiguity between low- and high-confidence samples. Module-level performance depended on the direction of the continuous module score. With classical defined as the positive class, modules with higher scores in basal-like tumors produced AUCs below 0.5. This reflects reversed score orientation rather than class imbalance. Expressing the same ranking with basal-like as the positive class yields 1−AUC and does not represent a separate model or an improvement in performance. Nevertheless, a mapping between image features and genes for the classical and basal cohorts could be derived from whole-slide-image morphology. This presents a novel means of holistically mapping physical features in pathology images to gene coexpression. The proportions of classical and basal patients are shown in Table 10.

**Table 7:** Module-level AUCs for Morphology-RNA LIONESS networks as an analysis of performance results for morphology ablations.

Module	No. genes	AUC
module_0	26	0.7496
module_1	19	0.3294

**Table 8:** Subtype classification AUCs for global RNA and Morphology-RNA network conditions as an ablation analysis at the global level using gene degree times mean gene expression as a module score.

Condition	Subtype AUC
RNA	0.6168
MORPH_RNA	0.5798

**Table 9:** Subtype discrimination by RT-RNA network modules using LIONESS-based connectivity. For each module, a patient-specific score was computed as the mean edge weight among all intramodule edges in that patient’s RT-RNA LIONESS network.

Module	Number of genes	AUC (classical as positive)	AUC (basal as positive)^a^
module_1	17	0.42	0.58
module_0	20	0.37	0.63

^a^Computed as $\text{1}-{\text{AUC}}_{\text{classical}}$ to reflect discrimination in the basal direction.

**Table 10:** Distribution of Moffitt subtypes in the TCGA PAAD cohort. Class imbalance may affect the uncertainty and calibration of performance estimates but does not determine whether AUC is above or below 0.5.

Subtype	Number of cases	Proportion of cohort (%)
Basal	110	60.1
Classical	73	39.9

The Morphology RNA-networks and the other networks serve as exploratory analysis of the contribution of each modality to the individual target. Using the same Moffitt gene space, the RNA-only selected-expression-module pipeline tested whether the mean RNA expression of genes selected within each training fold could discriminate subtype in held-out patients; it achieved a mean held-out AUC of 0.815 (Standard Deviation of 0.080; 95% Confidence Intervals, 0.716–0.914) under nested cross-validation. Direct logistic regression using the fixed 44-gene Moffitt set tested the conventional RNA-expression baseline without module selection and achieved a mean held-out AUC of 0.855 (SD 0.123; 95% CI, 0.702–1.000). The mean paired difference was 0.040 AUC in favor of logistic regression (95% CI, −0.046 to 0.126; paired fold-level p = 0.264). Selected RNA modules contained a mean of 22.4 genes (SD 1.9; range, 20–25); this assessed the reproducibility of RNA-module selection, with 18 of 29 selected genes retained in every fold and a mean pairwise module overlap of 0.780 (SD 0.062).

Under nested cross-validation, the patient-specific RT–RNA LIONESS intramodule-edge model tested whether mean integrated RT–RNA edge weights within each selected module could discriminate subtype in held-out patients; it achieved a mean AUC of 0.467 (SD 0.066; 95% CI, 0.386–0.549), indicating no reliable held-out subtype discrimination. The selected RT–RNA modules contained a mean of 19.8 genes (range, 19–21), with 15 of 24 genes retained in all five outer folds; this measured stable RT–RNA module selection rather than predictive performance. These AUCs were threshold-free and calculated from continuous logistic-regression probabilities, rather than from a fixed positive–negative classification threshold.

In exploratory global-network ablation analyses, RNA-only networks were constructed from rank-based RNA-expression correlations and scored using degree-weighted RNA expression (AUC, 0.629). RT-only networks were constructed from rank-based replication-timing-proxy correlations and scored using degree-weighted replication-timing-proxy values (AUC, 0.619). Joint RT–RNA networks combined RNA and replication-timing-proxy correlations in the edge weights and were scored using degree-weighted RNA expression (AUC, 0.636). Shuffling tested the robustness of network structure to null and parameter perturbations, rather than subtype-prediction accuracy. Overall, the RNA expression-module signal was substantially stronger under held-out validation, whereas the RT–RNA LIONESS analysis showed that replication timing altered patient-specific network structure and yielded stable modules without improving this static subtype-classification task.

## Discussion

Network biology provides a means of understanding gene coexpression based on previously characterized gene functions. By examining coexpression and clustering strength, gene-expression patterns can sometimes be inferred from genes with stronger or more central positions within a cluster, consistent with network theory and biological graph principles [1,2]. Spatial transcriptomics and digital pathology approaches typically integrate genes as large feature vectors, which may introduce noise if features are not appropriately thresholded or selected. Methods such as high-variance gene selection do not provide biological interpretability, while ontology-based selection does not always maximize predictive strength. Gene ontology can associate gene expression with morphological patterns across tissue regions, providing a means to infer expression across samples without directly re-estimating expression from morphological features.

Study-wise, individualized LIONESS network scores performed worse than global RNA-expression and worse than fixed-gene logistic summaries for held-out subtype classification. Network differences across patients were robust relative to the null-shuffle networks in terms of transitivity, density, and connectivity. In some instances, Individual modules concentrate subtype-associated signal, whereas global scores aggregate the full gene space and dilute that signal; moreover, direct whole-cohort module AUCs are exploratory because module discovery and evaluation use the same cohort, so they do not estimate held-out generalizability. This can provide a means to study patient-specific multimodal correlational networks and their relationship to subtype-associated gene-coexpression behavior. It can also be concluded that the mean based RNA-only global network, performed similarly to patient-specific RT-RNA LIONESS module scores as shown in Table 3 and Table 5. Certain subsets of Moffitt genes, and their correlations, were more indicative of subtype than others. It can also be concluded that the RT–RNA LIONESS intramodule-edge scores performed worse than the RNA expression-module scores when provided to regressions. However, these exploratory module-based results do not establish held-out improvement. The RNA expression-module regression performance was not significantly different from logistic-regression baselines relying on gene expression alone. The system of edge selections provides a means to identify gene modules and subsets that have stronger transcriptional relationships between tumor subtypes, although conclusions are limited by the small sample size. Compared to conventional subtyping systems such as single sample gene enrichment analysis (ssGEA) or clustering based assortment, this method provides a means to identify gene modules or subsets to classify patients using averages, similar to ssGEA, while also clustering gene modules by coexpression.

Furthermore, RT–RNA integration did not improve held-out subtype performance, whereas morphology–RNA analyses showed exploratory topology changes. Morphology-influenced RNA networks tended to show bifurcations between sets of genes, where more correlated genes became more strongly correlated and formed two clusters. RT-influenced RNA networks altered edge structure by creating new edges, pruning edges, or strengthening weakly correlated edges. These networks showed robust network properties under shuffling, but did not improve held-out subtype performance. These multimodal networks offer a means to understand changes in gene coexpression without requiring bijective mapping between modality-specific graphs. In turn, this offers a means to understand how gene networks change at the patient level when morphology and other modalities influence correlational weights. Future work involves using these edge-influenced multimodal networks to study changes in network structure with differences in protein expression, copy-number variants, and miRNA expression. Network analysis using principal eigenvalues and eigenvector centrality may further clarify process-level information. Additionally, different edge formulations can be used to model gene–gene interactions more accurately.

Network biology in the replication-timing domain would provide additional insight into how gene expression is influenced by chromatin structure and temporal replication programs [3–5,8]. The accuracy of these models depends primarily on the strength of correlations among genes rather than the specific numerical metrics used. Genetic networks therefore hold potential for quantifying and correlating expression across biological structures while incorporating the replication-timing dimension [3,5,8].

Although the methylation-based replication timing proxy may not improve subtyping accuracy, it improves robustness by emphasizing genes with strong regulatory signals. The methylation-based replication timing proxy has also been shown to stabilize network properties such as density and transitivity, consistent with long-range epigenetic organization and replication-domain structure [7, 9]. This suggests that gene expression can be modeled with respect to chromatin stability and genome-regulatory architecture.

Morphological embeddings also demonstrated strong capacity to detect and score modules corresponding to Basal-Like and Classical subtypes, aligning with previous findings on Pancreatic Adenocarcinoma (PDAC) subtype structure and morphology-linked differentiation states [10,11]. This supports modeling morphology alongside gene expression. Future biological networks may integrate protein cascades and morphological networks, while chromatin-based features and mutations operate as “invisible variables” that indirectly influence cascade behavior.

In digital pathology, multimodal models are increasingly necessary to associate slides with biological mechanisms that are not directly visible in raw histology [3,5,12]. By connecting morphological features to gene expression, models can learn to infer biological cascades from variations in cell neighborhoods, cell phenotypes, and patch-level motifs.

### Limitations

Partial-correlation networks were not constructed at the patient level, whereas LIONESS networks were constructed [12]. AUC is not strictly dependent on edge strength; it also depends on which genes are included in the modules influenced by morphological inputs. Increasing the number of nodes or genes lowers network resolution. The Search Tool for The Retrieving of Interacting Genes/Proteins (STRING) version 12.0 (https://string-db.org) database connections were attempted; however, connections among many Moffitt genes were incomplete or absent, as expected for sparse or poorly annotated regulatory interactions [17,18]. The global networks were not subtype-specific at the patient level but could characterize cohort-level variance related to tumor progression. Nodes were not constructed directly from morphology or methylation-based replication timing proxying; instead, these modalities influenced edge weights and module scores.

Other major limitations include the use of a single public retrospective cohort, lack of external validation, limited sample size, and possible overfitting. The indirect nature of methylation-derived replication-timing proxies provides an indirect lens but is not adequate in handling partially methylated domains and is not resolute at the gene-level. There is uncertainty about generalizability to routine clinical pathology specimens, especially cross-institution. There is substantial ambiguity resulting from low sample size, which can result in potential confounding from tumor purity, stromal content, batch effects and sampling heterogeneity. While the split between basal and classical patients was fair (65:35), there is question as to whether patients that are not well defined on either subtype influence model performance.

## Conclusions

Patient-specific methylation-based replication-timing-proxy-based correlative networks provide insight into how chromatin structure shapes gene-expression variation across patients [3–5,8]. This approach can help characterize cohort-level differences between classical and basal-like populations [10,11]. Although methylation-based replication timing did not improve predictive performance, it provided an effective means of approximating chromatin stability at the case level. Morphology-influenced networks demonstrated that image embeddings and phenotypes were meaningfully connected to PDAC subtype structure [10, 11]. Vision Transformer embeddings and other image-derived features can therefore be used to understand gene coexpression directly. Future work will include modeling downstream molecular consequences of gene expression, including protein-cascade signaling, in relation to morphological features.

## References

[1] Newman MEJ. Networks: an introduction. Oxford: Oxford University Press; 2010.

[2] Watts DJ, Strogatz SH. Collective dynamics of “small-world” networks. Nature. 1998;393(6684):440–2. doi: 10.1038/30918 9623998

[3] Rivera-Mulia JC, Gilbert DM. Replication timing and transcriptional control: beyond cause and effect—part II. Curr Opin Genet Dev. 2016;37:106–13. doi: 10.1016/j.gde.2016.03.002 PMC2677117 19345088

[4] Rhind N, Gilbert DM. DNA replication timing. Cold Spring Harb Perspect Biol. 2013;5(8):a010132. doi: 10.1101/cshperspect.a010132 23838440 PMC3721284

[5] Hansen RS, Thomas S, Sandstrom R, Canfield TK, Thurman RE, Weaver M, et al. Sequencing newly replicated DNA reveals widespread plasticity in human replication timing. Proc Natl Acad Sci U S A. 2010;107(1):139–44. doi: 10.1073/pnas.0912402107 19966280 PMC2806781

[6] Zhou W, Triche TJ Jr, Laird PW, Shen H. SeSAMe: reducing artifactual detection of DNA methylation by Infinium BeadChips in genomic deletions. Nucleic Acids Res. 2018;46(20):e123. doi: 10.1093/nar/gky691 30085201 PMC6237738

[7] Fortin J-P, Hansen KD. Reconstructing A/B compartments as revealed by Hi-C using long-range correlations in epigenetic data. Genome Biol. 2015;16(1):180. doi: 10.1186/s13059-015-0741-y 26316348 PMC4574526

[8] Mukherjee S, Callegari AJ. How replication timing shapes genome maintenance and cancer genome evolution. Front Cell Dev Biol. 2021;9:671120. doi: 10.3389/fcell.2021.671120

[9] Pope BD, Ryba T, Dileep V, Yue F, Wu W, Denas O, et al. Topologically associating domains are stable units of replication-timing regulation. Nature. 2014;515(7527):402–5. doi: 10.1038/nature13986 25409831 PMC4251741

[10] Moffitt RA, Marayati R, Flate EL, Volmar KE, Loeza SGH, Hoadley KA, et al. Virtual microdissection identifies distinct tumor- and stroma-specific subtypes of pancreatic ductal adenocarcinoma. Nat Genet. 2015;47(10):1168–78. doi: 10.1038/ng.3398 26343385 PMC4912058

[11] Rashid NU, Peng XL, Jin C, Moffitt RA, Volmar KE, Belt BA, et al. Purity independent subtyping of tumors (purist), a clinically robust, single-sample classifier for tumor subtyping in pancreatic cancer. Clin Cancer Res. 2020;26(1):82–92. doi: 10.1158/1078-0432.CCR-19-1467 31754050 PMC6942634

[12] Kuijjer ML, Tung MG, Yuan G, Quackenbush J, Glass K. Estimating Sample-Specific Regulatory Networks. iScience. 2019;14:226–40. doi: 10.1016/j.isci.2019.03.021 30981959 PMC6463816

[13] Shetty AV, Steer CJ, Low WC. Single cell multiomics approach to analyze replication timing and gene expression in mouse preimplantation embryos. Commun Biol. 2025;8(1):1245. doi: 10.1038/s42003-025-08694-5 40830419 PMC12365258

[14] Jaccard P. Étude comparative de la distribution florale dans une portion des Alpes et des Jura. Bull Soc Vaudoise Sci Nat. 1901;37:547–79.

[15] Good P. Permutation, parametric, and bootstrap tests of hypotheses. 3rd ed. New York: Springer; 2005.

[16] Efron B, Tibshirani RJ. An introduction to the bootstrap. Boca Raton: CRC Press. 1994.

[17] Marbach D, Costello JC, Küffner R, Vega NM, Prill RJ, Camacho DM, et al. Wisdom of crowds for robust gene network inference. Nat Methods. 2012;9(8):796–804. doi: 10.1038/nmeth.2016 22796662 PMC3512113

[18] Glass K, Huttenhower C, Quackenbush J, Yuan G-C. Passing messages between biological networks to refine predicted interactions. PLoS One. 2013;8(5):e64832. doi: 10.1371/journal.pone.0064832 23741402 PMC3669401

