# Supplementary material for: Patient-specific integration of RNA expression, methylation-derived replication-timing proxies, and histomorphology for pancreatic cancer subtyping
Source: PLoS One. 2026 Sep 15;21(9):e0358246. doi: 10.1371/journal.pone.0358246 (PMC13577548; doi:10.1371/journal.pone.0358246)
Supplement: Supplementary file 1 [file pone.0358246.s001.docx]

This study uses the TCGA-PAAD cohort to integrate RNA expression, methylation-derived replication-timing proxies, and pathology image embeddings into individualized LIONESS co-expression networks for basal-like and classical subtyping of pancreatic ductal adenocarcinoma. The topic is interesting, and the multimodal network approach is potentially useful. However, several key aspects of the study design, methodology, validation, and interpretation require clarification. I therefore recommend major revision.

Major comments

1. The sample selection and data-matching process are not sufficiently described. The authors should clearly report the final number of cases included for RNA, methylation, and pathology image analyses, how missing data were handled, and how molecular data were matched with image embeddings. A workflow diagram would be helpful.

2. The replication-timing feature is inferred from methylation data rather than directly measured. The biological validity of this proxy should be better justified or externally supported. Otherwise, the authors should use more cautious wording, such as “methylation-derived replication-timing proxy,” throughout the manuscript.

3. The reported model performance is not clearly presented. The abstract highlights module-level AUCs of approximately 0.80 and 0.75, whereas the overall subtype classification AUCs in the main text appear much lower. The authors should clearly distinguish module-level performance from overall model performance and avoid overstatement. Confidence intervals, cross-validation details, and comparisons with simpler RNA-based models should be added.

The study lacks independent validation. Since the analysis is based on a single TCGA cohort and involves feature selection, module construction, and parameter tuning, overfitting is a concern. The authors should clarify how information leakage was avoided and consider external validation or rigorous nested cross-validation.

4. Several methodological details need correction or expansion. For example, it is unclear whether partial correlation was used, and the reported replication-timing bin size appears inconsistent. The pathology image-processing pipeline, including slide selection, tumor region identification, patch extraction, quality control, and feature aggregation, should also be described in more detail.

Minor comments

1. The title is lengthy and could be shortened.

2. The claim of being the “first study” should be used cautiously.

3. Some figures are difficult to read because of small labels and low resolution.

4. The manuscript would benefit from careful English editing.
