# Supplementary material for: Patient-specific integration of RNA expression, methylation-derived replication-timing proxies, and histomorphology for pancreatic cancer subtyping
Source: PLoS One. 2026 Sep 15;21(9):e0358246. doi: 10.1371/journal.pone.0358246 (PMC13577548; doi:10.1371/journal.pone.0358246)
Supplement: Supplementary file 2 [file pone.0358246.s004.docx]

# Response to Reviewers

We thank the reviewers for their careful evaluation of our revised manuscript and constructive comments. The manuscript has been revised accordingly, as detailed below. All page and line references correspond to the revised manuscript.

## Reviewer #1

#### Comment:

“I have reviewed the revised manuscript and the authors' responses. The methodology is sound, the data support the conclusions, and the overall presentation has been substantially improved. I recommend acceptance in its current form.”

#### Response:

We thank the reviewer for their careful evaluation and positive assessment of the revised manuscript. We are pleased that the previous concerns have been adequately addressed.

## Reviewer #2

We thank the reviewer for these constructive comments, which have improved the clarity, technical transparency, and reproducibility of the manuscript.

### Minor Comment 1

“The change in the correlation threshold used for the exploratory global-network analysis should be explained a little more clearly.

The revised manuscript indicates that an absolute correlation threshold of 0.2 was used for the global degree-score analysis, whereas a threshold of 0.5 was used in other analyses to identify more strongly correlated gene subsets. Since the choice of threshold can influence network density, gene degree, and consequently the derived AUC values, it would be helpful for the authors to briefly state whether the 0.2 threshold was defined a priori based on methodological considerations or was selected during exploratory analysis.

No additional optimization analysis is necessarily required, but a short explanation of the rationale for using different thresholds would improve transparency and make the workflow easier to reproduce.”

#### Response:

We revised the threshold explanation to clarify that 0.2 was selected as a priori for the exploratory global degree score analysis to retain the maximum number of valid degree values and examine how a broader set of lower weight edges affected degree weighted patient scores and model performance. The manuscript contrasts this exploratory threshold with the 0.5 threshold used in the remaining analyses to isolate more strongly correlated gene subsets. The revision also explains that retaining additional lower weight edges can alter patient scores nonuniformly because the graph conditions differ in their edge weight distributions and edge counts.

**Location:** Page 19, lines 295 to 304.

### Minor Comment 2

“The nested cross-validation procedure is now much clearer and appears to appropriately separate training and held-out data. However, given the relatively complex sequence of gene selection, network construction, module detection, module selection, and logistic-regression fitting, I suggest adding a concise statement or schematic description confirming that all data-dependent preprocessing and feature/module selection steps were repeated independently within each outer-training partition.

The authors already describe the major safeguards against information leakage, so this is mainly a request for presentation rather than a request for additional analysis. A short workflow summary in the Methods or the existing diagram would make the validation strategy easier for readers to understand and reproduce.”

#### Response:

We added a concise workflow summary stating that feature and module selection steps were repeated independently within each training partition. The selected gene module and fitted regression were then evaluated on the held out outer test fold. The outer test fold was not used for module construction or tuning. This clarification makes the separation between training and held out evaluation explicit.

**Location:** Page 12, lines 203 to 225.

### Minor Comment 3

“Given the computational nature of this study, reproducibility would be further improved by making the main analysis scripts or code used for network construction, LIONESS analysis, module selection, and nested cross-validation publicly available, if possible.

The underlying TCGA data are publicly available, but the analytical pipeline contains several study-specific processing and parameter-selection steps. Providing the relevant scripts through a public repository, together with software/package versions and the principal analysis parameters, would make it substantially easier for other investigators to reproduce the reported results.”

#### Response:

The analysis code is publicly available at <https://github.com/Alejandro21236/LIONESS4PATH>. The repository contains scripts for network construction, LIONESS analysis, module selection, morphology based analyses, and nested cross validation. It also includes analysis labels, shell scripts containing the principal parameters, the package environment YAML file, and setup instructions. The large morphology embeddings and processed RNA sequencing matrices are not duplicated in the repository because of their storage requirements and the availability of the underlying source data through TCGA.

**Location:** Page 32, lines 521 to 523.

## Reviewer #3

We thank the reviewer for their careful assessment and constructive recommendations.

### Comment 1

“In the Abstract, when 'LIONESS' and 'TCGA' first appear, if they are abbreviations, please provide their full names. In addition, the Abstract is somewhat lengthy and should be appropriately condensed. Starting from the Introduction, all abbreviations should also be spelled out upon first use.”

#### Response:

We defined Linear Interpolation to Obtain Network Estimates for Single Samples (LIONESS) and The Cancer Genome Atlas (TCGA) upon their first appearances in the Abstract. The Abstract was also condensed by removing redundant background and methodological details.

**Location:** Page 1, line 10 through Page 2, line 28.

### Comment 2

“Please revise the language for clarity and fluency. For instance, the phrasing 'structure-how genes interact-remains' could be reworded to sound more natural and smoother in English.”

#### Response:

We revised the cited sentence to read 'genetic coexpression structure remains underexplored.' We also revised additional sentences for clarity, including the descriptions of the nested cross validation procedure and network conditions.

**Location:** Page 3, lines 58 to 59. Additional revisions appear throughout the manuscript.

### Comment 3

“In the Limitations section, the 'STRING database' is mentioned, but no specific version is provided. Please authors include the version number used and the corresponding URL link for easy access by readers.”

#### Response:

We added the full name of STRING, specified version 12.0, and provided the corresponding URL: [https://string-db.org](https://string-db.org/).

**Location:** Page 30, lines 483 to 485.

### Comment 4

“The font size of the axis labels in all figures appears too small. Please authors increase the font size appropriately to improve readability.”

#### Response:

We enlarged and reorganized the displayed figure panels to improve the effective sizes of axis titles, tick labels, and category labels at the final manuscript display size. Figure 1 was also reformatted so that its individual panels are displayed at a larger size without changing the underlying analysis.

**Location:** Figures 1 to 6, Pages 14 to 23.

### Comment 5

“In each figure legend, the specific biological groups represented in the figure should be clearly stated, and the statistical methods used should also be indicated.”

#### Response:

We added a clarification stating that all network conditions were constructed using the complete matched TCGA PAAD study cohort and were not stratified by subtype. The panels primarily compare computational network conditions rather than separately plotted biological groups. We also defined the network condition abbreviations, including RNA, RT, RT-RNA, RNA-PCORR, RT-RNA-PCORR, MORPH-RNA, GRID, and SHUFFLE.

The figure legends identify the network metrics and analytical comparisons displayed, including Jaccard similarity where applicable. Most panels present descriptive network properties rather than inferential comparisons. Figure 6 explicitly states that no inferential hypothesis test was applied to its descriptive summaries.

**Location:** Page 13, lines 234 to 240, and the legends for Figures 1 to 6 on Pages 14 to 23.

### Comment 6

“The current labels for the subpanels (e.g., (a), (b), (c)) are not sufficiently standardized. For example, '(a) Module match to RT_RNA top: RNA' should be revised; please keep only the sequential labels (a), (b), (c), etc., and move all explanatory details to the figure legends.”

#### Response:

We standardized the labels beneath all multipart figure panels so that they contain only the sequential labels (a), (b), (c), and so forth. The corresponding explanatory panel descriptions are provided in the figure legends.

**Location:** Figures 1 to 6, Pages 14 to 23.

### Comment 7

“Table 6 is currently split into two small tables, which hampers reading continuity. Please merge them into a single complete table, or alternatively, place it as a supplementary table.”

#### Response:

Table 6 has been formatted as one continuous table with a repeated header when it continues onto the following page. The previous visual division resulted from the table crossing a page boundary rather than from an intentional separation of the data.

**Location:** Pages 20 to 21, beginning at line 324.
