# Supplementary material for: Patient-specific integration of RNA expression, methylation-derived replication-timing proxies, and histomorphology for pancreatic cancer subtyping
Source: PLoS One. 2026 Sep 15;21(9):e0358246. doi: 10.1371/journal.pone.0358246 (PMC13577548; doi:10.1371/journal.pone.0358246)
Supplement: Peer review history

# Peer review: Decision Letter 0

**DOI:** 10.1371/journal.pone.0358246.r001

**Authors:** Kangkang Ji

14 Jul 2026

Dear Dr. Leyva,

Thank you for submitting your manuscript to PLOS ONE. After careful consideration, we feel that it has merit but does not fully meet PLOS ONE’s publication criteria as it currently stands. Therefore, we invite you to submit a revised version of the manuscript that addresses the points raised during the review process.

Please submit your revised manuscript by Aug 28 2026 11:59PM. If you will need more time than this to complete your revisions, please reply to this message or contact the journal office at plosone@plos.org. When you're ready to submit your revision, log on to https://www.editorialmanager.com/pone/ and select the 'Submissions Needing Revision' folder to locate your manuscript file.

- A letter that responds to each point raised by the academic editor and reviewer(s). You should upload this letter as a separate file labeled 'Response to Reviewers'.
- A marked-up copy of your manuscript that highlights changes made to the original version. You should upload this as a separate file labeled 'Revised Manuscript with Track Changes'.
- An unmarked version of your revised paper without tracked changes. You should upload this as a separate file labeled 'Manuscript'.

If you would like to make changes to your financial disclosure, please include your updated statement in your cover letter. Guidelines for resubmitting your figure files are available below the reviewer comments at the end of this letter.

If applicable, we recommend that you deposit your laboratory protocols in protocols.io to enhance the reproducibility of your results. Protocols.io assigns your protocol its own identifier (DOI) so that it can be cited independently in the future. For instructions see: https://journals.plos.org/plosone/s/submission-guidelines#loc-laboratory-protocols. Additionally, PLOS ONE offers an option for publishing peer-reviewed Lab Protocol articles, which describe protocols hosted on protocols.io. Read more information on sharing protocols at https://plos.org/protocols?utm_medium=editorial-email&utm_source=authorletters&utm_campaign=protocols.

As the corresponding author, your ORCID iD is verified in the submission system and will appear in the published article. PLOS supports the use of ORCID, and we encourage all coauthors to register for an ORCID iD and use it as well. Please encourage your coauthors to verify their ORCID iD within the submission system before final acceptance, as unverified ORCID iDs will not appear in the published article. *Only*  the individual author can complete the verification step; PLOS staff *cannot*  verify ORCID iDs on behalf of authors.

We look forward to receiving your revised manuscript.

Kind regards,

Kangkang Ji, Ph.D., M.D.

Academic Editor

PLOS One

Journal Requirements:

When submitting your revision, we need you to address these additional requirements.

1. Please ensure that your manuscript meets PLOS ONE's style requirements, including those for file naming. The PLOS ONE style templates can be found at

https://journals.plos.org/plosone/s/file?id=wjVg/PLOSOne_formatting_sample_main_body.pdf and

https://journals.plos.org/plosone/s/file?id=ba62/PLOSOne_formatting_sample_title_authors_affiliations.pdf

2. Thank you for stating the following financial disclosure:

"The project described was supported in part by R01 CA276301 (PIs: Niazi and Chen) from the National Cancer Institute, Pelatonia under IRP CC13702 (PIs: Niazi, Vilgelm, and Roy), The Ohio State University Department of Pathology and Comprehensive Cancer Center. The content is solely the responsibility of the authors and does not necessarily represent the official views of the National Cancer Institute or National Institutes of Health or The Ohio State University."

Please state what role the funders took in the study.  If the funders had no role, please state: "The funders had no role in study design, data collection and analysis, decision to publish, or preparation of the manuscript."

If this statement is not correct you must amend it as needed.

Please include this amended Role of Funder statement in your cover letter; we will change the online submission form on your behalf.

3.  Please note that your Data Availability Statement is currently missing a direct link to access each database. If your manuscript is accepted for publication, you will be asked to provide these details on a very short timeline. We therefore suggest that you provide this information now, though we will not hold up the peer review process if you are unable.

4. If the reviewer comments include a recommendation to cite specific previously published works, please review and evaluate these publications to determine whether they are relevant and should be cited. There is no requirement to cite these works unless the editor has indicated otherwise.

[Note: HTML markup is below. Please do not edit.]

Reviewers' comments:

Reviewer's Responses to Questions

**Comments to the Author**

1. Is the manuscript technically sound, and do the data support the conclusions?

Reviewer #1: Yes

Reviewer #2: Partly

**********

2. Has the statistical analysis been performed appropriately and rigorously?

Reviewer #1: Yes

Reviewer #2: No

**********

3. Have the authors made all data underlying the findings in their manuscript fully available?

The PLOS Data policy

Reviewer #1: Yes

Reviewer #2: Yes

**********

4. Is the manuscript presented in an intelligible fashion and written in standard English?

Reviewer #1: Yes

Reviewer #2: No

**********

Reviewer #1: This study uses the TCGA-PAAD cohort to integrate RNA expression, methylation-derived replication-timing proxies, and pathology image embeddings into individualized LIONESS co-expression networks for basal-like and classical subtyping of pancreatic ductal adenocarcinoma. The topic is interesting, and the multimodal network approach is potentially useful. However, several key aspects of the study design, methodology, validation, and interpretation require clarification. I therefore recommend major revision.

Major comments

1. The sample selection and data-matching process are not sufficiently described. The authors should clearly report the final number of cases included for RNA, methylation, and pathology image analyses, how missing data were handled, and how molecular data were matched with image embeddings. A workflow diagram would be helpful.

2. The replication-timing feature is inferred from methylation data rather than directly measured. The biological validity of this proxy should be better justified or externally supported. Otherwise, the authors should use more cautious wording, such as “methylation-derived replication-timing proxy,” throughout the manuscript.

3. The reported model performance is not clearly presented. The abstract highlights module-level AUCs of approximately 0.80 and 0.75, whereas the overall subtype classification AUCs in the main text appear much lower. The authors should clearly distinguish module-level performance from overall model performance and avoid overstatement. Confidence intervals, cross-validation details, and comparisons with simpler RNA-based models should be added.

The study lacks independent validation. Since the analysis is based on a single TCGA cohort and involves feature selection, module construction, and parameter tuning, overfitting is a concern. The authors should clarify how information leakage was avoided and consider external validation or rigorous nested cross-validation.

4. Several methodological details need correction or expansion. For example, it is unclear whether partial correlation was used, and the reported replication-timing bin size appears inconsistent. The pathology image-processing pipeline, including slide selection, tumor region identification, patch extraction, quality control, and feature aggregation, should also be described in more detail.

Minor comments

1. The title is lengthy and could be shortened.

2. The claim of being the “first study” should be used cautiously.

3. Some figures are difficult to read because of small labels and low resolution.

4. The manuscript would benefit from careful English editing.

Reviewer #2: The study addresses an interesting and clinically relevant topic. Pancreatic ductal adenocarcinoma remains a highly lethal malignancy, and improved integration of molecular subtype information with histomorphology and other biological features may be valuable. The attempt to combine RNA expression, methylation-derived replication-timing proxies, morphology-based embeddings, and individualized network analysis is potentially innovative.

However, from a clinical reader’s perspective, the current manuscript remains difficult to interpret and requires substantial revision before its scientific and clinical value can be adequately assessed. My recommendation is major revision.

1. The AUC results are inconsistent across the abstract, main text, and tables.

The abstract states that the RNA-replication-timing model achieved an AUC of approximately 80%, and that morphology-based networks achieved an AUC of approximately 75%. However, the results reported in the tables are not consistent with this overall interpretation. For example, Table 2 reports RNA and RT_RNA subtype AUCs close to 0.54 and 0.53, respectively, while Table 8 reports a Morph_RNA subtype AUC of approximately 0.58. Table 9 also reports RT_RNA module AUCs that differ from the values emphasized in the abstract.

It appears that some of the stronger AUCs may refer to selected modules rather than the overall predictive model. This distinction is important. The authors should clearly define the primary performance endpoint and state whether each AUC refers to the full model, a selected module, a cross-validated result, or an exploratory analysis. The abstract should reflect the main validated findings rather than emphasizing only the best-performing module.

2. The statistical interpretation of AUC and class imbalance needs correction.

The manuscript suggests that AUC values below 0.5 may be explained by the higher proportion of basal-like cases in the cohort. This explanation is not statistically appropriate. Class imbalance can affect accuracy, precision, calibration, and the uncertainty of performance estimates, but it does not by itself cause AUC to fall below 0.5. An AUC below 0.5 usually suggests that the direction of the score is reversed, that the positive class has been defined inconsistently, or that the classifier is not discriminating in the intended direction.

The authors should revise this interpretation and clearly state which subtype is treated as the positive class for each AUC calculation. If values are converted using 1 − AUC, this should be explained consistently and carefully.

3. Some conclusions appear stronger than the data currently support.

Several statements in the abstract, discussion, and conclusion imply that replication-timing proxies reveal clinically relevant regulatory structure, replication stress, chromatin accessibility profiles, or therapeutic response relevance. These are interesting hypotheses, but the current study appears to be based mainly on retrospective TCGA data and indirect methylation-derived replication-timing proxies. There is no direct replication-timing assay, no independent experimental validation, and no clinical treatment-response validation.

The authors should moderate these claims. The findings should be presented as exploratory and hypothesis-generating unless additional validation is provided. From a clinical perspective, it is especially important to avoid implying immediate therapeutic or diagnostic relevance without stronger supporting evidence.

4. The Discussion should be expanded and made more specific to the study findings.

The current Discussion remains relatively general and does not sufficiently explain the study’s own results. In particular, the authors should discuss why the overall model performance appears low while selected module-level AUCs are higher. This distinction is clinically important, because a selected module does not necessarily represent a robust or generalizable predictive model.

The authors should also discuss whether the overlap with PURIST genes represents an independent finding or is partly expected from the way the subtype labels and gene sets were defined. A clearer comparison with established PDAC transcriptomic subtyping methods would help readers understand the added value of the proposed approach.

5. The limitations section should be substantially expanded.

Several limitations should be more clearly acknowledged. These include the use of a single public retrospective cohort, lack of external validation, limited sample size, possible overfitting or module-selection bias, the indirect nature of methylation-derived replication-timing proxies, and uncertainty about generalizability to routine clinical pathology specimens.

The authors should also discuss potential confounding from tumor purity, stromal content, sampling heterogeneity, batch effects, and the possibility that morphology-based embeddings may capture non-tumor or technical features unless tumor-region selection and quality control are clearly described.

6. The figures require substantial improvement.

Several figures are difficult to read. Font sizes are small, panel labels are not sufficiently clear, and some multi-panel figures appear visually crowded. Figure 6 is particularly difficult to interpret because many diagnostic plots are compressed into one figure. Some figures look more like direct pipeline outputs than publication-ready scientific figures.

The authors should redesign the main figures using larger fonts, clearer panel labels, simplified layouts, and more informative legends. Exploratory or diagnostic plots may be better placed in supplementary materials.

7. Reference order and formatting should be corrected.

The reference numbering appears to be out of order. For example, early citations in the Introduction do not appear to follow the order of the reference list. The authors should carefully check all in-text citations and the reference list to ensure that references are numbered and formatted according to journal requirements.

8. The title should be revised.

The current title is difficult to read and contains unnatural phrasing. In particular, “Personalized Morphology” and “subtyping genes” are not clear expressions. A more concise and natural title would improve readability. For example, the authors may consider a title such as:

“Patient-Specific Integration of RNA Expression, Methylation-Derived Replication-Timing Proxies, and Histomorphology for Pancreatic Cancer Subtyping.”

9. The English language, tables, and manuscript formatting require careful revision.

There are multiple grammatical errors, typographical errors, and awkward phrases throughout the manuscript. Examples include “exhibitory gene expression,” “where as,” “similiarity,” “condtions,” “publically,” and “wholistically.” Some methods and results sentences are difficult to follow. In addition, phrases such as “Values are taken exactly as provided in the CSV” are not appropriate for a polished scientific manuscript.

The manuscript would benefit from thorough scientific English editing and careful formatting of tables, figure captions, references, and headings.

Overall assessment:

The study concept is interesting and may be relevant to pancreatic cancer subtyping. However, the current manuscript requires major revision. The most important revisions are to reconcile the inconsistent AUC results, correct the interpretation of AUC and class imbalance, moderate claims that go beyond the data, expand the Discussion and limitations, improve figure readability, correct reference order, revise the title, and substantially improve the English language and formatting.

**********

what does this mean?). If published, this will include your full peer review and any attached files.

If you choose “no”, your identity will remain anonymous but your review may still be made public.

**Do you want your identity to be public for this peer review?**  For information about this choice, including consent withdrawal, please see our Privacy Policy

Reviewer #1: No

Reviewer #2: No

**********

[NOTE: If reviewer comments were submitted as an attachment file, they will be attached to this email and accessible via the submission site. Please log into your account, locate the manuscript record, and check for the action link "View Attachments". If this link does not appear, there are no attachment files.]

To ensure your figures meet our technical requirements, please review our figure guidelines: https://journals.plos.org/plosone/s/figures

You may also use PLOS’s free figure tool, NAAS, to help you prepare publication quality figures: https://journals.plos.org/plosone/s/figures#loc-tools-for-figure-preparation.

NAAS will assess whether your figures meet our technical requirements by comparing each figure against our figure specifications.

Attachment Submitted filename: Reviewer_Comments_PONE-D-26-13963.docx

# Author comment: Author response to Decision Letter 1

**DOI:** 10.1371/journal.pone.0358246.r002

4 Aug 2026

Dear Editor and Reviewers:

We thank the Editor and Reviewers for their detailed and constructive evaluation. We have revised the manuscript to clarify cohort matching, the methylation-derived replication-timing proxy, network construction, image processing, and the distinction between exploratory analyses and the primary nested cross-validation endpoint. The revised manuscript also includes confidence intervals, explicit leakage safeguards, updated figures and tables, a revised title, sequential Vancouver-style references, and expanded limitations and discussion.

All manuscript locations below refer to the revised clean manuscript and its continuous line numbering.

Reviewer #1

Major Comment 1. “The sample selection and data-matching process are not sufficiently described. The authors should clearly report the final number of cases included for RNA, methylation, and pathology image analyses, how missing data were handled, and how molecular data were matched with image embeddings. A workflow diagram would be helpful.”

Response: We clarified the final analytic cohort and matching procedure. The manuscript now states that 183 TCGA-PAAD cases were subtyped and matched to methylation proxies and pathology embeddings at a 1:1 case-to-embedding ratio. It also describes preprocessing, overlap-based alignment, and the case-level mapping of slide embeddings. Diagram 1 was added to show the multimodal workflow and the training/held-out evaluation sequence. Revised manuscript: pp. 5–6, lines 92–126; pp. 11–12, lines 192–223; Diagram 1.

Summary: Cohort matching, multimodal alignment, and the workflow diagram were added and clarified.

Major Comment 2. “The replication-timing feature is inferred from methylation data rather than directly measured. The biological validity of this proxy should be better justified or externally supported. Otherwise, the authors should use more cautious wording, such as “methylation-derived replication-timing proxy,” throughout the manuscript.”

Response: We revised the terminology throughout to describe this feature as a methylation-derived replication-timing proxy rather than direct replication timing. The Methods now explains inversion, z-scoring, smoothing, and domain-level aggregation, and the Discussion, Limitations, and Conclusions retain the proxy language and do not equate the inferred measure with a direct replication-timing assay. Revised manuscript: pp. 1–2, lines 10–35; pp. 6–7, lines 113–126; pp. 28–29, lines 459–475.

Summary: The inferred feature is consistently described as a methylation-derived replication-timing proxy.

Major Comment 3. “The reported model performance is not clearly presented. The abstract highlights module-level AUCs of approximately 0.80 and 0.75, whereas the overall subtype classification AUCs in the main text appear much lower. The authors should clearly distinguish module-level performance from overall model performance and avoid overstatement. Confidence intervals, cross-validation details, and comparisons with simpler RNA-based models should be added. The study lacks independent validation. Since the analysis is based on a single TCGA cohort and involves feature selection, module construction, and parameter tuning, overfitting is a concern. The authors should clarify how information leakage was avoided and consider external validation or rigorous nested cross-validation.”

Response: We now explicitly distinguish exploratory module-level analyses from the primary model-performance endpoint. The primary endpoint is the mean held-out test AUC across a stratified five-fold outer nested cross-validation, with three stratified inner folds used exclusively within each outer-training set to select the candidate module. For each outer fold, the selected module score was used to fit logistic regression only in the outer-training patients, and performance was then evaluated once in the untouched outer-test fold. Thus, the reported mean AUC of 0.815 represents validated held-out performance of the RNA selected-expression-module pipeline, not the AUC of a single selected module or an in-sample analysis.

In contrast, the module-level AUCs reported in Tables 3–5 and 7–9 are now identified as exploratory direct whole-cohort analyses. These values describe the subtype association of individual mean-expression or LIONESS connectivity modules and are not presented as the performance of the overall validated model. Similarly, the global network ablation AUCs are described as exploratory global degree-score analyses rather than cross-validated classifier performance. The Abstract was revised to report the validated RNA selected-expression-module result (mean held-out AUC, 0.815; SD, 0.080; 95% CI, 0.716–0.914) and the fixed 44-gene Moffitt logistic-regression comparator (mean held-out AUC, 0.855; SD, 0.123; 95% CI, 0.702–1.000), with no significant paired fold-level difference (mean difference, 0.040; p = 0.264).

To prevent information leakage, candidate-gene selection, network construction, module detection, inner-fold module selection, and logistic-regression fitting were performed using only the relevant outer-training data. The outer-test fold was never used for model selection, tuning, or fitting. The lack of independent external validation remains explicitly acknowledged as a limitation of this single-cohort study.

Revised manuscript: pp. 1–2, lines 24–35; pp. 12–13, lines 209–226; pp. 23–24, lines 351–380; p. 28, lines 459–466.

Summary: The manuscript now separates exploratory individual-module and global-network AUCs from the primary five-outer-fold/three-inner-fold nested-CV endpoint, reports uncertainty and the RNA baseline comparison, and explicitly documents safeguards against information leakage.

Major Comment 4. “Several methodological details need correction or expansion. For example, it is unclear whether partial correlation was used, and the reported replication-timing bin size appears inconsistent. The pathology image-processing pipeline, including slide selection, tumor region identification, patch extraction, quality control, and feature aggregation, should also be described in more detail.”

Response: We expanded the methodological description and parameter table. We also corrected the replication-timing bin-size inconsistency: all methylation-derived replication-timing proxies were calculated using 200-kb domains, not 300-kb domains; the previous 300-kb reference was a typographical error. We apologize for this inconsistency, which has now been corrected throughout the manuscript.

The revised Methods specify the Spearman-correlation threshold, top-KKK edge filtering, RT shuffles, bootstrap edge-stability analysis, and the role of the partial-correlation control. The pathology pipeline is now described in detail, including surgical-resection slide selection, HEST tissue segmentation and artifact removal, extraction of 256 × 256 patches, pretrained UNIv2 encoding through Trident, slide-level embedding aggregation, case-ID matching with molecular data, standardization, and incorporation of morphology into network edge weights and patient-specific module scores.

Revised manuscript: pp. 6–10, lines 113–188; pp. 11–12, lines 192–206.

Summary: The replication-timing domain size was corrected to 200 kb, and the network, partial-correlation, and morphology-processing methods were expanded.

Minor Comment 1. “The title is lengthy and could be shortened.”

Response: The title was shortened and revised to use direct, standard terminology for the modalities and PDAC subtyping. Revised manuscript: p. 1, lines 1–2.

Summary: Title shortened.

Minor Comment 2. “The claim of being the “first study” should be used cautiously.”

Response: We qualified the novelty statement with “To our knowledge” and limited it to the specific multimodal individualized-network integration examined here. Revised manuscript: p. 1, lines 17–23.

Summary: Novelty claim qualified.

Minor Comment 3. “Some figures are difficult to read because of small labels and low resolution.”

Response: The figures were revised for larger labels, clearer panel lettering, improved resolution, and more readable layouts. Diagnostic panels retained in the main manuscript are those used to support the reported topology, stability, and modality-integration analyses. Revised manuscript: Figs. 1–6, pp. 13–22.

Summary: Figures redesigned for readability.

Minor Comment 4. “The manuscript would benefit from careful English editing.”

Response: The manuscript text, tables, headings, equations, and captions were revised for consistency and readability. Scientific terminology and nomenclature were also standardized across the Abstract, Methods, Results, Discussion, and tables. Revised manuscript: throughout.

Summary: Language and manuscript presentation revised throughout.

Reviewer #2

Comment 1. “The AUC results are inconsistent across the abstract, main text, and tables. The abstract states that the RNA-replication-timing model achieved an AUC of approximately 80%, and that morphology-based networks achieved an AUC of approximately 75%. However, the results reported in the tables are not consistent with this overall interpretation. For example, Table 2 reports RNA and RT_RNA subtype AUCs close to 0.54 and 0.53, respectively, while Table 8 reports a Morph_RNA subtype AUC of approximately 0.58. Table 9 also reports RT_RNA module AUCs that differ from the values emphasized in the abstract. It appears that some of the stronger AUCs may refer to selected modules rather than the overall predictive model. This distinction is important. The authors should clearly define the primary performance endpoint and state whether each AUC refers to the full model, a selected module, a cross-validated result, or an exploratory analysis. The abstract should reflect the main validated findings rather than emphasizing only the best-performing module.”

Response: We resolved the apparent inconsistency by separating exploratory, full-cohort analyses from the primary validated endpoint. The Abstract now reports the nested-CV RNA selected-expression-module result (mean held-out AUC 0.815) and its conventional logistic-regression comparator, rather than the previous selected-module values. Tables 2–9 are retained as exploratory analyses and their text identifies whether each result is a global-network score, a mean module score, or a patient-specific LIONESS connectivity score. The Results now explicitly reports the held-out RT–RNA LIONESS result and distinguishes it from direct whole-cohort module AUCs. Revised manuscript: pp. 1–2, lines 24–35; pp. 18–23, lines 290–350; pp. 23–24, lines 351–380.

Summary: Exploratory AUCs are separated from the validated nested-CV endpoint.

Comment 2. “The statistical interpretation of AUC and class imbalance needs correction. The manuscript suggests that AUC values below 0.5 may be explained by the higher proportion of basal-like cases in the cohort. This explanation is not statistically appropriate. Class imbalance can affect accuracy, precision, calibration, and the uncertainty of performance estimates, but it does not by itself cause AUC to fall below 0.5. An AUC below 0.5 usually suggests that the direction of the score is reversed, that the positive class has been defined inconsistently, or that the classifier is not discriminating in the intended direction. The authors should revise this interpretation and clearly state which subtype is treated as the positive class for each AUC calculation. If values are converted using 1 − AUC, this should be explained consistently and carefully.”

Response: We revised the statistical interpretation of AUC and explicitly distinguish the exploratory table-specific analyses from the primary validated endpoint. Classical is defined as the positive class unless otherwise stated. An AUC below 0.5 is now interpreted as a reversal of score orientation toward basal-like cases, not as an effect of class imbalance. Where basal-positive AUCs are reported, they are calculated as 1−AUC from the same continuous score ranking and are presented only to clarify direction; they do not represent a separate model or improved performance.

We also clarify why the RNA value of approximately 0.54 in Table 2 differs from the Abstract’s AUC of 0.815. Table 2 is an exploratory global-network degree-score analysis, not a cross-validated classifier. For each global condition, a gene’s degree was defined as the number of retained network edges incident on that gene after correlation filtering and top-K edge selection. Each patient’s score was then calculated as the sum of each gene’s degree multiplied by that patient’s corresponding RNA-expression value (or replication-timing-proxy value for the RT-only condition). The direct whole-cohort AUC in Table 2 therefore tests whether global network connectivity, used as a degree-weighted molecular summary, is associated with subtype. It does not test the selected-module logistic-regression model and does not use held-out patients. Here, “global” means that one network is constructed from the full cohort of available patients for a given modality condition, rather than constructing a separate network for each patient. Each global edge represents the cohort-level correlation or integrated similarity between a pair of genes. The resulting global graph is then used to calculate gene degree, identify modules, or summarize network topology. In contrast, LIONESS generates a separate patient-specific network by comparing the full-cohort graph with the corresponding graph recalculated after that patient is excluded.

In Table 2, we identified two technical sources of the earlier discrepancy. First, the original global degree-score analysis used an absolute edge threshold of 0.5, whereas the revised analysis uses the intended threshold of 0.2; the more permissive threshold retains moderate gene–gene correlations, changes gene degrees, and therefore produces revised degree-weighted patient scores and higher exploratory AUCs. This was needed to show the performance of each modality with the maximum number of valid edges, rather than using only the highly correlated subsets used to isolate gene modules for the following module based analysis. The RT-only NaN value reflected an earlier exploratory output and was corrected in the revised analysis. The updated Table 2 reports the finite RT result generated from the final aligned data-processing pipeline.

Tables 3 and 4 are also exploratory. They evaluate whether the mean expression or mean replication-timing-proxy value of each module from a global network is associated with subtype across the same cohort. These analyses were used to identify modules with stronger subtype-associated aggregate signals and to localize where signal is concentrated; they are not estimates of overall model generalizability.

Tables 5, 7, and 9 test a different question: whether patient-specific LIONESS intramodule connectivity is associated with subtype. For each patient, the module score is the mean personalized edge weight among gene pairs within that module. These direct whole-cohort LIONESS AUCs are exploratory evaluations of subtype-associated individualized network structure, rather than nested cross-validated predictive performance. Table 8 similarly reports an exploratory global degree-weighted score for the morphology–RNA condition.

By contrast, the Abstract’s AUC of 0.815 is the primary validated result: the mean held-out AUC from the five-outer-fold/three-inner-fold nested-CV RNA selected-expression-module pipeline. In each outer fold, module selection occurred only within the outer-training patients, logistic regression was fitted only in the outer-training patients, and the AUC was calculated once in the untouched outer-test patients. Thus, the Table 2 value of approximately 0.54 and the Abstract value of 0.815 measure different quantities and are no longer presented as competing estimates of the same model.

Revised manuscript: pp. 12–13, lines 217–226; pp. 18–23, lines 290–350; pp. 23–24, lines 351–380.

Summary: The manuscript now explains AUC orientation and class definition, identifies Tables 2–9 as exploratory analyses of global degree scores, mean module v

# Peer review: Decision Letter 1

**DOI:** 10.1371/journal.pone.0358246.r003

**Authors:** Kangkang Ji

25 Aug 2026

Dear Dr. Leyva,

Thank you for submitting your manuscript to PLOS One. After careful consideration, we feel that it has merit but does not fully meet PLOS One’s publication criteria as it currently stands. Therefore, we invite you to submit a revised version of the manuscript that addresses the points raised during the review process.

Please submit your revised manuscript by Oct 09 2026 11:59PM. If you will need more time than this to complete your revisions, please reply to this message or contact the journal office at plosone@plos.org. When you're ready to submit your revision, log on to https://www.editorialmanager.com/pone/ and select the 'Submissions Needing Revision' folder to locate your manuscript file.

- A letter that responds to each point raised by the academic editor and reviewer(s). You should upload this letter as a separate file labeled 'Response to Reviewers'.
- A marked-up copy of your manuscript that highlights changes made to the original version. You should upload this as a separate file labeled 'Revised Manuscript with Track Changes'.
- An unmarked version of your revised paper without tracked changes. You should upload this as a separate file labeled 'Manuscript'.

If you would like to make changes to your financial disclosure, please include your updated statement in your cover letter. Guidelines for resubmitting your figure files are available below the reviewer comments at the end of this letter.

If applicable, we recommend that you deposit your laboratory protocols in protocols.io to enhance the reproducibility of your results. Protocols.io assigns your protocol its own identifier (DOI) so that it can be cited independently in the future. For instructions see: https://journals.plos.org/plosone/s/submission-guidelines#loc-laboratory-protocols. Additionally, PLOS One offers an option for publishing peer-reviewed Lab Protocol articles, which describe protocols hosted on protocols.io. Read more information on sharing protocols at https://plos.org/protocols?utm_medium=editorial-email&utm_source=authorletters&utm_campaign=protocols.

As the corresponding author, your ORCID iD is verified in the submission system and will appear in the published article. PLOS supports the use of ORCID, and we encourage all coauthors to register for an ORCID iD and use it as well. Please encourage your coauthors to verify their ORCID iD within the submission system before final acceptance, as unverified ORCID iDs will not appear in the published article. *Only*  the individual author can complete the verification step; PLOS staff *cannot*  verify ORCID iDs on behalf of authors.

We look forward to receiving your revised manuscript.

Kind regards,

Kangkang Ji, Ph.D., M.D.

Academic Editor

PLOS One

Journal Requirements:

If the reviewer comments include a recommendation to cite specific previously published works, please review and evaluate these publications to determine whether they are relevant and should be cited. There is no requirement to cite these works unless the editor has indicated otherwise.

Please review your reference list to ensure that it is complete and correct. If you have cited papers that have been retracted, please include the rationale for doing so in the manuscript text, or remove these references and replace them with relevant current references. Any changes to the reference list should be mentioned in the rebuttal letter that accompanies your revised manuscript. If you need to cite a retracted article, indicate the article’s retracted status in the References list and also include a citation and full reference for the retraction notice.

[Note: HTML markup is below. Please do not edit.]

Reviewers' comments:

Reviewer's Responses to Questions

**Comments to the Author**

Reviewer #1: All comments have been addressed

Reviewer #2: (No Response)

Reviewer #3: All comments have been addressed

**********

2. Is the manuscript technically sound, and do the data support the conclusions?

Reviewer #1: Yes

Reviewer #2: Yes

Reviewer #3: Yes

**********

3. Has the statistical analysis been performed appropriately and rigorously?

Reviewer #1: Yes

Reviewer #2: Yes

Reviewer #3: I Don't Know

**********

4. Have the authors made all data underlying the findings in their manuscript fully available?

The PLOS Data policy

Reviewer #1: Yes

Reviewer #2: Yes

Reviewer #3: Yes

**********

5. Is the manuscript presented in an intelligible fashion and written in standard English?

Reviewer #1: Yes

Reviewer #2: Yes

Reviewer #3: Yes

**********

Reviewer #1: I have reviewed the revised manuscript and the authors' responses. The methodology is sound, the data support the conclusions, and the overall presentation has been substantially improved. I recommend acceptance in its current form.

Reviewer #2: The manuscript is technically impeccable, and I believe the current analysis largely supports the conclusions. I only have a few minor issues left to resolve without requiring extensive additional analysis.

I have only a few remaining minor comments that I believe can be addressed without substantial additional analysis.

Minor Comment 1

The change in the correlation threshold used for the exploratory global-network analysis should be explained a little more clearly.

The revised manuscript indicates that an absolute correlation threshold of 0.2 was used for the global degree-score analysis, whereas a threshold of 0.5 was used in other analyses to identify more strongly correlated gene subsets. Since the choice of threshold can influence network density, gene degree, and consequently the derived AUC values, it would be helpful for the authors to briefly state whether the 0.2 threshold was defined a priori based on methodological considerations or was selected during exploratory analysis.

No additional optimization analysis is necessarily required, but a short explanation of the rationale for using different thresholds would improve transparency and make the workflow easier to reproduce.

Minor Comment 2

The nested cross-validation procedure is now much clearer and appears to appropriately separate training and held-out data. However, given the relatively complex sequence of gene selection, network construction, module detection, module selection, and logistic-regression fitting, I suggest adding a concise statement or schematic description confirming that all data-dependent preprocessing and feature/module selection steps were repeated independently within each outer-training partition.

The authors already describe the major safeguards against information leakage, so this is mainly a request for presentation rather than a request for additional analysis. A short workflow summary in the Methods or the existing diagram would make the validation strategy easier for readers to understand and reproduce.

Minor Comment 3

Given the computational nature of this study, reproducibility would be further improved by making the main analysis scripts or code used for network construction, LIONESS analysis, module selection, and nested cross-validation publicly available, if possible.

The underlying TCGA data are publicly available, but the analytical pipeline contains several study-specific processing and parameter-selection steps. Providing the relevant scripts through a public repository, together with software/package versions and the principal analysis parameters, would make it substantially easier for other investigators to reproduce the reported results.

Reviewer #3: Dear authors,

Your manuscript entitled “Patient-specific integration of RNA expression, methylation-derived replication-timing proxies, and histomorphology for pancreatic cancer subtyping” (Manuscript ID: PONE-D-26-13963R1) indicates that this study, using a multimodal LIONESS network, integrates replication‑timing proxies and morphological features within the same framework, thereby enabling the identification of reproducible gene modules and providing in‑depth exploration of subtype‑related dynamic changes in biological connectivity. The research topic is of significant scientific importance and considerable novelty. Nevertheless, before the manuscript can be published, several issues require further revision and clarification:

(1) In the Abstract, when “LIONESS” and “TCGA” first appear, if they are abbreviations, please provide their full names. In addition, the Abstract is somewhat lengthy and should be appropriately condensed. Starting from the Introduction, all abbreviations should also be spelled out upon first use.

(2) Please revise the language for clarity and fluency. For instance, the phrasing “structure—how genes interact—remains” could be reworded to sound more natural and smoother in English.

(3) In the Limitations section, the “STRING database” is mentioned, but no specific version is provided. Please authors include the version number used and the corresponding URL link for easy access by readers.

(4) The font size of the axis labels in all figures appears too small. Please authors increase the font size appropriately to improve readability.

(5) In each figure legend, the specific biological groups represented in the figure should be clearly stated, and the statistical methods used should also be indicated.

(6) The current labels for the subpanels (e.g., (a), (b), (c)) are not sufficiently standardized. For example, “(a) Module match to RT_RNA top: RNA” should be revised; please keep only the sequential labels (a), (b), (c), etc., and move all explanatory details to the figure legends.

(7) Table 6 is currently split into two small tables, which hampers reading continuity. Please merge them into a single complete table, or alternatively, place it as a supplementary table.

**********

what does this mean?). If published, this will include your full peer review and any attached files.

If you choose “no”, your identity will remain anonymous but your review may still be made public.

**Do you want your identity to be public for this peer review?**  For information about this choice, including consent withdrawal, please see our Privacy Policy

Reviewer #1: No

Reviewer #2: No

Reviewer #3: No

**********

[NOTE: If reviewer comments were submitted as an attachment file, they will be attached to this email and accessible via the submission site. Please log into your account, locate the manuscript record, and check for the action link "View Attachments". If this link does not appear, there are no attachment files.]

To ensure your figures meet our technical requirements, please review our figure guidelines: https://journals.plos.org/plosone/s/figures

You may also use PLOS’s free figure tool, NAAS, to help you prepare publication quality figures: https://journals.plos.org/plosone/s/figures#loc-tools-for-figure-preparation.

NAAS will assess whether your figures meet our technical requirements by comparing each figure against our figure specifications.

# Author comment: Author response to Decision Letter 2

**DOI:** 10.1371/journal.pone.0358246.r004

27 Aug 2026

Response to Reviewers

We thank the reviewers for their careful evaluation of our revised manuscript and constructive comments. The manuscript has been revised accordingly, as detailed below. All page and line references correspond to the revised manuscript.

Reviewer #1

Comment:

“I have reviewed the revised manuscript and the authors' responses. The methodology is sound, the data support the conclusions, and the overall presentation has been substantially improved. I recommend acceptance in its current form.”

Response:

We thank the reviewer for their careful evaluation and positive assessment of the revised manuscript. We are pleased that the previous concerns have been adequately addressed.

Reviewer #2

We thank the reviewer for these constructive comments, which have improved the clarity, technical transparency, and reproducibility of the manuscript.

Minor Comment 1

“The change in the correlation threshold used for the exploratory global-network analysis should be explained a little more clearly.

The revised manuscript indicates that an absolute correlation threshold of 0.2 was used for the global degree-score analysis, whereas a threshold of 0.5 was used in other analyses to identify more strongly correlated gene subsets. Since the choice of threshold can influence network density, gene degree, and consequently the derived AUC values, it would be helpful for the authors to briefly state whether the 0.2 threshold was defined a priori based on methodological considerations or was selected during exploratory analysis.

No additional optimization analysis is necessarily required, but a short explanation of the rationale for using different thresholds would improve transparency and make the workflow easier to reproduce.”

Response:

We revised the threshold explanation to clarify that 0.2 was selected as a priori for the exploratory global degree score analysis to retain the maximum number of valid degree values and examine how a broader set of lower weight edges affected degree weighted patient scores and model performance. The manuscript contrasts this exploratory threshold with the 0.5 threshold used in the remaining analyses to isolate more strongly correlated gene subsets. The revision also explains that retaining additional lower weight edges can alter patient scores nonuniformly because the graph conditions differ in their edge weight distributions and edge counts.

Location: Page 19, lines 295 to 304.

Minor Comment 2

“The nested cross-validation procedure is now much clearer and appears to appropriately separate training and held-out data. However, given the relatively complex sequence of gene selection, network construction, module detection, module selection, and logistic-regression fitting, I suggest adding a concise statement or schematic description confirming that all data-dependent preprocessing and feature/module selection steps were repeated independently within each outer-training partition.

The authors already describe the major safeguards against information leakage, so this is mainly a request for presentation rather than a request for additional analysis. A short workflow summary in the Methods or the existing diagram would make the validation strategy easier for readers to understand and reproduce.”

Response:

We added a concise workflow summary stating that feature and module selection steps were repeated independently within each training partition. The selected gene module and fitted regression were then evaluated on the held out outer test fold. The outer test fold was not used for module construction or tuning. This clarification makes the separation between training and held out evaluation explicit.

Location: Page 12, lines 203 to 225.

Minor Comment 3

“Given the computational nature of this study, reproducibility would be further improved by making the main analysis scripts or code used for network construction, LIONESS analysis, module selection, and nested cross-validation publicly available, if possible.

The underlying TCGA data are publicly available, but the analytical pipeline contains several study-specific processing and parameter-selection steps. Providing the relevant scripts through a public repository, together with software/package versions and the principal analysis parameters, would make it substantially easier for other investigators to reproduce the reported results.”

Response:

The analysis code is publicly available at https://github.com/Alejandro21236/LIONESS4PATH. The repository contains scripts for network construction, LIONESS analysis, module selection, morphology based analyses, and nested cross validation. It also includes analysis labels, shell scripts containing the principal parameters, the package environment YAML file, and setup instructions. The large morphology embeddings and processed RNA sequencing matrices are not duplicated in the repository because of their storage requirements and the availability of the underlying source data through TCGA.

Location: Page 32, lines 521 to 523.

Reviewer #3

We thank the reviewer for their careful assessment and constructive recommendations.

Comment 1

“In the Abstract, when 'LIONESS' and 'TCGA' first appear, if they are abbreviations, please provide their full names. In addition, the Abstract is somewhat lengthy and should be appropriately condensed. Starting from the Introduction, all abbreviations should also be spelled out upon first use.”

Response:

We defined Linear Interpolation to Obtain Network Estimates for Single Samples (LIONESS) and The Cancer Genome Atlas (TCGA) upon their first appearances in the Abstract. The Abstract was also condensed by removing redundant background and methodological details.

Location: Page 1, line 10 through Page 2, line 28.

Comment 2

“Please revise the language for clarity and fluency. For instance, the phrasing 'structure-how genes interact-remains' could be reworded to sound more natural and smoother in English.”

Response:

We revised the cited sentence to read 'genetic coexpression structure remains underexplored.' We also revised additional sentences for clarity, including the descriptions of the nested cross validation procedure and network conditions.

Location: Page 3, lines 58 to 59. Additional revisions appear throughout the manuscript.

Comment 3

“In the Limitations section, the 'STRING database' is mentioned, but no specific version is provided. Please authors include the version number used and the corresponding URL link for easy access by readers.”

Response:

We added the full name of STRING, specified version 12.0, and provided the corresponding URL: https://string-db.org.

Location: Page 30, lines 483 to 485.

Comment 4

“The font size of the axis labels in all figures appears too small. Please authors increase the font size appropriately to improve readability.”

Response:

We enlarged and reorganized the displayed figure panels to improve the effective sizes of axis titles, tick labels, and category labels at the final manuscript display size. Figure 1 was also reformatted so that its individual panels are displayed at a larger size without changing the underlying analysis.

Location: Figures 1 to 6, Pages 14 to 23.

Comment 5

“In each figure legend, the specific biological groups represented in the figure should be clearly stated, and the statistical methods used should also be indicated.”

Response:

We added a clarification stating that all network conditions were constructed using the complete matched TCGA PAAD study cohort and were not stratified by subtype. The panels primarily compare computational network conditions rather than separately plotted biological groups. We also defined the network condition abbreviations, including RNA, RT, RT-RNA, RNA-PCORR, RT-RNA-PCORR, MORPH-RNA, GRID, and SHUFFLE.

The figure legends identify the network metrics and analytical comparisons displayed, including Jaccard similarity where applicable. Most panels present descriptive network properties rather than inferential comparisons. Figure 6 explicitly states that no inferential hypothesis test was applied to its descriptive summaries.

Location: Page 13, lines 234 to 240, and the legends for Figures 1 to 6 on Pages 14 to 23.

Comment 6

“The current labels for the subpanels (e.g., (a), (b), (c)) are not sufficiently standardized. For example, '(a) Module match to RT_RNA top: RNA' should be revised; please keep only the sequential labels (a), (b), (c), etc., and move all explanatory details to the figure legends.”

Response:

We standardized the labels beneath all multipart figure panels so that they contain only the sequential labels (a), (b), (c), and so forth. The corresponding explanatory panel descriptions are provided in the figure legends.

Location: Figures 1 to 6, Pages 14 to 23.

Comment 7

“Table 6 is currently split into two small tables, which hampers reading continuity. Please merge them into a single complete table, or alternatively, place it as a supplementary table.”

Response:

Table 6 has been formatted as one continuous table with a repeated header when it continues onto the following page. The previous visual division resulted from the table crossing a page boundary rather than from an intentional separation of the data.

Location: Pages 20 to 21, beginning at line 324.

Attachment Submitted filename: Minor_Revision_Res.docx

# Editor report: Decision Letter 2

**DOI:** 10.1371/journal.pone.0358246.r005

**Authors:** Kangkang Ji

30 Aug 2026

Patient-specific integration of RNA expression, methylation-derived replication-timing proxies, and histomorphology for pancreatic cancer subtyping

PONE-D-26-13963R2

Dear Dr. Leyva,

We’re pleased to inform you that your manuscript has been judged scientifically suitable for publication and will be formally accepted for publication once it meets all outstanding technical requirements.

Within one week, you’ll receive an e-mail detailing the required amendments. When these have been addressed, you’ll receive a formal acceptance letter and your manuscript will be scheduled for publication.

An invoice will be generated when your article is formally accepted. Please note, if your institution has a publishing partnership with PLOS and your article meets the relevant criteria, all or part of your publication costs will be covered. Please make sure your user information is up-to-date by logging into Editorial Manager at Editorial Manager® and clicking the ‘Update My Information' link at the top of the page. For questions related to billing, please contact billing support.

If your institution or institutions have a press office, please notify them about your upcoming paper to help maximize its impact. If they’ll be preparing press materials, please inform our press team as soon as possible -- no later than 48 hours after receiving the formal acceptance. Your manuscript will remain under strict press embargo until 2 pm Eastern Time on the date of publication. For more information, please contact onepress@plos.org.

Kind regards,

Kangkang Ji, Ph.D., M.D.

Academic Editor

PLOS One

Additional Editor Comments (optional):

Reviewers' comments:

# Editor report: Acceptance letter

**DOI:** 10.1371/journal.pone.0358246.r006

**Authors:** Kangkang Ji

PONE-D-26-13963R2

PLOS One

Dear Dr. Leyva,

I'm pleased to inform you that your manuscript has been deemed suitable for publication in PLOS One. Congratulations! Your manuscript is now being handed over to our production team.

At this stage, our production department will prepare your paper for publication. This includes ensuring the following:

* All references, tables, and figures are properly cited

* All relevant supporting information is included in the manuscript submission,

* There are no issues that prevent the paper from being properly typeset

You will receive further instructions from the production team, including instructions on how to review your proof when it is ready. Please keep in mind that we are working through a large volume of accepted articles, so please give us a few days to review your paper and let you know the next and final steps.

Lastly, if your institution or institutions have a press office, please let them know about your upcoming paper now to help maximize its impact. If they'll be preparing press materials, please inform our press team within the next 48 hours. Your manuscript will remain under strict press embargo until 2 pm Eastern Time on the date of publication. For more information, please contact onepress@plos.org.

You will receive an invoice from PLOS for your publication fee after your manuscript has reached the completed accept phase. If you receive an email requesting payment before acceptance or for any other service, this may be a phishing scheme. Learn how to identify phishing emails and protect your accounts at https://explore.plos.org/phishing.

If we can help with anything else, please email us at customercare@plos.org.

Thank you for submitting your work to PLOS One and supporting open access.

Kind regards,

PLOS One Editorial Office Staff

on behalf of

Dr. Kangkang Ji

Academic Editor

PLOS One
